# Platelet-derived circRNAs signature in patients with gastroenteropancreatic neuroendocrine tumors

**DOI:** 10.1186/s12967-023-04417-8

**Published:** 2023-08-16

**Authors:** Federica Campolo, Franz Sesti, Tiziana Feola, Giulia Puliani, Antongiulio Faggiano, Maria Grazia Tarsitano, Marta Tenuta, Valeria Hasenmajer, Elisabetta Ferretti, Monica Verrico, Daniele Gianfrilli, Mary Anna Venneri, Andrea M. Isidori, Elisa Giannetta

**Affiliations:** 1 Department of Experimental Medicine, Sapienza University of Rome, Rome, Italy; 2 Neuroendocrinology, Neuromed Institute, IRCCS, Pozzilli, Italy; 3 Oncological Endocrinology Unit, IRCCS Regina Elena National Cancer Institute, Rome, Italy; 4 Department of Clinical and Molecular Medicine, Sant’Andrea Hospital, ENETS Center of Excellence, Sapienza University of Rome, Rome, Italy; 5 Department of Medical and Surgical Science, Magna Graecia University, Catanzaro, Italy; 6 UOC Endocrinology, Metabolic Diseases, Andrology SMIC08, Policlinico Umberto I, Rome, Italy; 7 Department of Radiological, Oncological and Anatomo-Pathological Sciences, Sapienza University of Rome, Rome, Italy; 8 Centre for Rare Diseases (ENDO-ERN Accredited), Policlinico Umberto I, Rome, Italy

**Keywords:** Neuroendocrine tumors (NETs), Tumor-educated platelets (TEPs), Circular RNAs (circRNAs), Liquid biopsy, Biomarkers

## Abstract

**Background:**

Neuroendocrine tumors (NETs) early diagnosis is a clinical challenge that require a deep understanding of molecular and genetic features of this heterogeneous group of neoplasms. However, few biomarkers exist to aid diagnosis and to predict prognosis and treatment response. In the oncological field, tumor-educated platelets (TEPs) have been implicated as central players in the systemic and local responses to tumor growth, thereby altering tumor specific RNA profile. Although TEPs have been found to be enriched in RNAs, few studies have investigated the potential of a type of RNA, circular RNAs (circRNA), as platelet-derived biomarkers for cancer. In this proof-of-concept study, we aim to demonstrate whether the circRNAs signature of tumor educated platelets can be used as a liquid biopsy biomarker for the detection of gastroenteropancreatic (GEP)-NETs and the prediction of the early response to treatment.

**Methods:**

We performed a 24-months, prospective proof-of-concept study in men and women with histologically proven well-differentiated G1-G2 GEP-NET, aged 18–80 years, naïve to treatment. We performed a RNAseq analysis of circRNAs obtained from TEPs samples of 10 GEP-NETs patients at baseline and after 3 months from therapy (somatostatin analogs or surgery) and from 5 patients affected by non-malignant endocrinological diseases enrolled as a control group.

**Results:**

Statistical analysis based on p < 0.05 resulted in the identification of 252 circRNAs differentially expressed between GEP-NET and controls of which 109 were up-regulated and 143 were down-regulated in NET patients. Further analysis based on an FDR value ≤ 0.05 resulted in the selection of 5 circRNAs all highly significant downregulated. The same analysis on GEP-NETs at baseline and after therapy in 5 patients revealed an average of 4983 remarkably differentially expressed circRNAs between follow-up and baseline samples of which 2648 up-regulated and 2334 down-regulated, respectively. Applying p ≤ 0.05 and FDR ≤ 0.05 filters, only 3/5 comparisons gave statistically significant results.

**Conclusions:**

Our findings identified for the first time a circRNAs signature from TEPs as potential diagnostic and predictive biomarkers for GEP-NETs.

**Supplementary Information:**

The online version contains supplementary material available at 10.1186/s12967-023-04417-8.

## Background

Neuroendocrine neoplasms (NENs) are a heterogeneous group of neoplasms, extremely variable in site of onset, biological features, clinical presentation, and course. The term NENs included the well-differentiated forms, also known as neuroendocrine tumors (NETs), and the poorly differentiated neoplasms, also known as neuroendocrine carcinomas (NECs) [1].

Non-functioning NET patients may suffer from latency in diagnosis due to the heterogeneity of tumor biology and the onset of non-specific symptoms that may have been present for many years [2]. In recent years, liquid biopsy has received growing attention for tumor detection, allowing for understanding what kind of genetic or molecular changes are taking place in a tumor [3], however its application in the NET field is limited. Currently, the most reliable prognostic markers used in clinical practice are tumor differentiation, grade, and stage [4]. Given NET’s heterogeneity in terms of clinical behavior and disease progression there is a medical need to rely on easy-to-execute and repeatable analysis, mostly circulating biomarkers, which can predict prognosis and early tumor response to treatment [5]. The discovery of new sensitive and specific diagnostic and predictive biomarkers is a crucial step toward improved clinical management of patients with NETs. Circular RNAs (circRNAs) belong to an evolutionarily conserved class of non-coding RNAs (ncRNAs) of the eukaryotic transcriptome, generated from back splicing of exons, introns or both [6]. They are historically able to regulate gene expression at transcriptional level by targeting different microRNA (miRNAs) and protein-coding genes [7]. In recent years, circRNAs become a hot topic due to their ability to regulate a plethora of biological processes aside from transcription, including translation, splicing and protein–protein interaction [8, 9].

All circRNAs showed peculiar features such as a long half-life in bloodstream compared to linear RNAs, and a high abundance and stability due to the absence of free 5ʹ and 3ʹ ends, all characteristics that make them stable and therefore potentially usable as a biomarker for cancer diagnosis and progression [10, 11].

Several studies suggested that circRNAs are enriched in platelets compared to nucleated cell types [12] and exhibited a powerful functional potential in regulating tumor proliferation, apoptosis and metastatization, suggesting that circRNAs may be promising target molecules in cancer [13]. Platelets are considered fundamental components of the tumor microenvironment actively contributing to tumor initiation, tumor progression, and therapy response [14]. Tumor-derived factors can “educate” platelets through several mechanisms including RNA or protein cargo transfer via exosomes, or receptor-mediated endocytosis of soluble factors within circulation [15]. Tumor-educated platelets (TEPs) are therefore able to take up tumor-derived secreted membrane vesicles which can contain tumor-associated RNAs that are distinct from healthy individuals [16]. Given the easiness to obtain and purify platelets, even from metastatic cancer patients, they emerged as promising biomarkers for cancer diagnosis and progression [17], and it is nowadays one of the possible approaches of liquid biopsies in cancers [18]. However, this innovative approach has never been transferred to the NENs field.

In this proof-of-concept study, we investigated whether circRNAs derived from TEPs may function as novel blood-based biomarkers for NET detection and early treatment response.

## Methods

### Study design

We performed a 24-months, prospective proof-of-concept study in men and women with the following inclusion criteria: histologically proven well-differentiated G1–G2 gastroenteropancreatic (GEP)-NET, age 18–80 years, naïve to treatment, belonging to our outpatient’s endocrinology clinic of the Department of Experimental Medicine at “Sapienza” University of Rome, in the Neuroendocrine Tumor task force Unit (NETTARE) of the “Policlinico Umberto I” University Hospital. Control group consists of patients affected by non-malignant endocrinological diseases, e.g., benign thyroid dysfunction, matched for age, gender, ethnicity and platelets count. Asymptomatic individuals had no medical history of diagnosis with any type of cancer prior to and/or at the moment of the blood collection. Exclusion criteria were severe chronic kidney disease (stage 4–5), clinical or laboratory signs of significant respiratory, cardiological, hematological and hepatobiliary disease, and other non-neuroendocrine malignancies. Blood sample was drawn at baseline defined as the time of the diagnosis before starting treatment according to clinical practice and current guidelines [19–21]. The study was performed in the “Policlinico Umberto I” University Hospital in Rome (Italy) and approved by the local ethics review board (5917), published on public registries (NCT04464122—REBORN Study) and conducted in accordance with the Declaration of Helsinki and good clinical practice. All patients provided written informed consent before enrolment. The trial was conducted between Sept 2020 and Sept 2022. This study adhered to the Strengthening the Reporting of Observational Studies in Epidemiology (STROBE) guidelines for reporting.

### Platelets isolation

Whole blood was collected in EDTA-coated purple-capped Vacutainer tubes (Becton Dickinson, Franklin Lakes, NJ, USA). Tubes were centrifuged at 120 ×*g* for 20 min at RT to separate platelet-rich plasma from nucleated blood cells. Plasma was then centrifuged at 360 ×*g* for 20 min at RT to pellet platelets. Platelet pellets were finally resuspended in RNAlater (Thermo Scientific, Waltham, MA, USA) and stored at − 80 °C until use.

### RNA extraction

Total RNA isolation was performed using miRNeasy Micro Kit (Qiagen, Hilden, Germany), according to the manufacturer’s instructions. On column DNase digestion was performed during extraction. RNA quality was assessed using RNA 6000 Picochip—Bioanalyzer 2100 (Agilent, Santa Clara, CA, USA). Only samples with a RIN-value greater than 7 and/or distinctive rRNA curves were included for analysis.

### Library construction

Library preparation was performed using SMARTer® Stranded Total RNA-Seq Kit v2—Pico Input Mammalian (Takara Bio Inc., Shiga, Japan) following manufacturer’s recommendations. Library quality and quantity were assessed with Qubit 2.0 DNA HS Assay as well as Tapestation D1000 Assay (Agilent, Santa Clara, CA, USA). Final libraries were then quantified using the QuantStudio^®^ 5 System (Applied Biosystems, California, USA) prior to equimolar pooling based on qPCR QC values.

### RNA Sequencing and data analysis

Sequencing was performed on an Illumina^®^ NovaSeq (Illumina, San Diego, CA, USA) with a read length configuration of 150 paired-ends for 120 M paired-ends reads (60 M in each direction) per sample. Run files were demultiplexed using bcl2fastq Software v2.20 (Illumina, San Diego, CA, USA). A quality check was performed on the raw data, removing low quality portions of NGS reads. The trimming step was performed with the following parameters: the minimum length was set to 35 bp and the quality score to 25 using the BBDuk Software. The quality before and after trimming was assessed with the software FASTQC.

HISAT-v2.1.0 was used to map the sequenced reads against an in-house generated from the University of California Santa Cruz (UCSC) reference, which is based on the human reference genome (hg38). After sorting for name and chromosome, followed by indexing with Samtools-v1.9, consistency, and quality of.bam files were checked using Integrative Genomics Viewer-v2.5.3.

### CircRNAs annotation and identification of differentially expressed circRNAs

CircRNAs annotation was carried out with CIRCexplorer2 Software. The high-quality reads were mapped on the human reference genome (hg38) using STAR. The chimeric reads were then processed with CIRCexplorer2 providing the official hg38 annotation (Ensembl release 105).

The identification of the differentially expressed circRNAs was performed with the package edgeR, the threshold for significance is FDR ≤ 0.05. Only the circRNAs with at least 3 reads in each replicate separated per group were considered. Three groups were analyzed: controls, GEP-NET baseline and GEP-NET follow-up. Each group consists of different biological replicates: 5 controls, 10 GEP-NET patients at baseline and 5 GEP-NET patients at follow-up.

A second step of annotation was performed via blast search against the circBase database [22] using the ‘List Search’ option at http://www.circbase.org/cgi-bin/listsearch.cgi. The blast search was conducted against the Homo sapiens genome version hg19.

Analysis of the differentially expressed circRNAs between NET patients and healthy donors and between NET patients baseline and NET patients follow-up was performed in R using the edgeR package [23].

### Gene ontology analysis

The functional annotation of the target mRNAs of differentially expressed circRNAs was performed by Gene Ontology (GO) term pathway analyses. Corrected p values with p ≤ 0.05 and FDR ≤ 0.05 were considered to indicate significant enrichment.

## Results

### Study population

From September 2020 to September 2022, 14 subjects with histologically proven G1-G2 NET were screened. Four patients (1 female, 3 males) were excluded because of entry criteria not met, 1 refused to participate, 10 entered the study (3 females and 7 males) median age 59.5 (interquartile range 57–63.25) (Table 1). All patients were European Caucasian. Concomitant disease and medications are listed in Table 1. Among the GEP-NET group, 5 patients were re-evaluated at an early follow up: three months after the treatment started according to good clinical practice. The remaining 5 patients did not require any medical or surgical treatment according to good clinical practice. Five healthy subjects (3 female, 2 males) were enrolled in the control group, median age 65 (interquartile range 45–67.5) (Table 1).

**Table 1:** Clinical and general characteristics of study population at baseline

Group	Age	Sex	Comorbidities	Smoking	Site	Functioning	Grade	Stage (TNM)
*Patients*								
#1	60	F	No	No	Pancreas	No	G1	Localized (cT1N0M0)
#2	59	F	No	No	Pancreas	Yes (insulinoma)	G1/G2	Metastatic (pT2pN0M1a R0)
#3	45	M	Autoimmune thyroiditis, hypothyroidism	No	Pancreas	Yes (insulinoma)	G1	Localized (pT1pN0)
#4	80	M	IFG, Hypertension, Dyslipidemia, MI	No	Ileum	No	G2	Metastatic
#5	61	F	DM, Hypertension, Dyslipidemia	No	Ileum	No	G2	Metastatic
#6	61	M	DM, Glaucoma, Cryptogenic Organizing Pneumonia, JAK2 +	No	Pancreas/Duodenum	No	G2	Metastatic
#7	57	M	No	No	Pancreas	Yes (insulinoma)	G2	Metastatic
#8	57	M	IFG, hypertension, dyslipidemia, stroke	Yes	Ileum	No	G2	Metastatic
#9	59	M	Hypertension, Thyroid nodule	No	Ileum	No	G1	Metastatic
#10	70	M	DM, Hypertension, Dyslipidemia, COPD, Hypothyroidism, MGUS	Yes	Pancreas	No	G1	Localized
*Controls*								
#1	66	M	Hypertension, multinodular goiter	No	–	–	–	–
#2	44	F	Multinodular goiter	No	–	–	–	–
#3	46	M	IFG, hypertension, thyroid nodule	No	–	–	–	–
#4	69	F	Multinodular goiter	No	–	–	–	–
#5	65	F	Hypertension	No	–	–	–	–

*F* female, *M* male, *MI* myocardial infarction, *IFG* impaired fasting glucose, *DM* diabetes mellitus type 2, *COPD* chronic obstructive pulmonary disease, *MGUS* monoclonal gammopathy of undetermined significance

### Profiling of platelets circRNA repertoire in GEP-NET patients vs controls

**Fig. 1:**
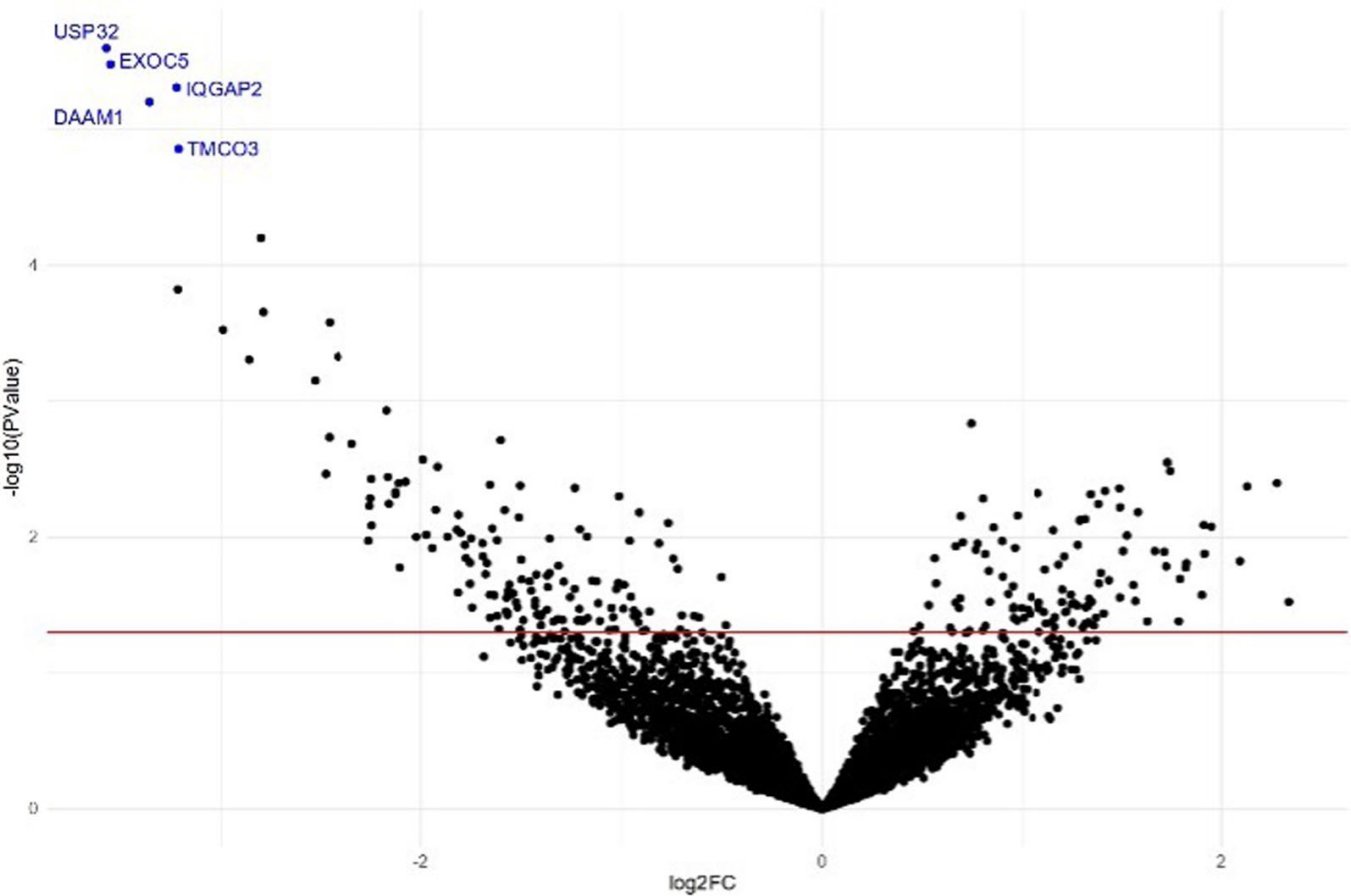
Volcano plot of differentially expressed circRNAs (Baseline NET patients *vs* Healthy donors). The negative log10 of the p-value is plotted on the y-axis, and the log2 of the FC is plotted on the x-axis. Red line marks p-value 0.05. Blue dots indicate downregulated circRNAs with p < 0.05 and FDR < 0.05

To investigate whether circRNAs could represent a potential non-invasive diagnostic biomarker for GEP-NET, we performed an RNA sequencing analysis of circRNAs of TEPs isolated from GEP-NET patients at baseline comparing their expression profile to that of control subjects. Structural and genomic features of circRNAs identified in this study are depicted in Additional file 1: Figure S1. We evaluated the performance of the analysis by checking for the presence of annotated platelets-specific circRNAs as positive controls (Additional file 1: Table S1). Our analysis revealed the presence of 36.339/67.295 annotated sequences in TEPs from GEP-NET samples and 25.712/48.514 annotated sequences in platelets from control samples. After processing of raw sequencing data, differentially expressed analysis of circRNAs was performed applying different statistical filters as cut-off. We were able to identify 5959 circRNAs in the compared sequenced samples (GEP-NET *vs* controls). Statistical filtering resulted in the identification of 252 circRNAs differentially expressed between GEP-NET and controls (p ≤ 0.05) of which 109 were up-regulated and 143 were down-regulated in cancer patients (Fig. 1 and Table 2).

**Table 2:** Statistical analysis on circRNA comparisons

Comparison	N° circRNAs	N° circRNAs with p ≤ 0.05			N° circRNAs with p ≤ 0.05 and FDR ≤ 0.05		
		Up	Down	Total	Up	Down	Total
*GEP-NETs vs controls*	**5959**	109	143	**252**	0	5	**5**
*GEP-NETs: baseline vs follow-up*							
* Patient #1*	**21908**	8491	2943	**11434**	4280	1482	**5762**
* Patient #2*	**21736**	6238	3031	**9269**	0	0	**0**
* Patient #3*	**24468**	2996	3242	**6238**	0	0	**0**
* Patient #4*	**22072**	3129	8053	**11182**	1269	2333	**3602**
* Patient #5*	**20893**	3434	6590	**10024**	2397	3188	**5585**

Table shows the total number of circRNAs identified for the comparison GEP-NETs at baseline *vs* healthy donors and GEP-NETs at baseline *vs* early follow-up and the number of up and downregulated circRNAs with p < 0.05 and p < 0.05 + FDR < 0.05

Bold values refer to total number of circRNAs. Bold was used to graphically emphatize total values in the table

Further analysis based on an FDR value ≤ 0.05 resulted in the selection of 5 circRNAs all highly significant downregulated: Ubiquitin Specific Peptidase 32 (*USP32*), Exocyst Complex Component 5 (*EXOC5*), IQ motif containing GTPase activating protein 2 (*IQGAP2*), Dishavelled Associated Activator Morphogenesis 1 (*DAAM1*), Transmembrane and Coiled-Coil Domains 3 (*TMCO3*). Genomic position and statistical significance of these circRNAs are shown in Table 3.

**Table 3:** Genomic position and statistical significance of circRNAs differentially expressed in GEP-NETs at baseline *vs* healthy donors’ comparison

Gene/strand/chr/genomic position	logFC	logCPM	LR	p value	FDR
USP32:−:17:60223410:60226231	− 3.58	4.04	22.14	0.0000025	0.00938
EXOC5:−:14:57229733:57237366	− 3.56	4.28	21.62	0.0000033	0.00938
IQGAP2: + :5:76592837:76606303	− 3.23	3.68	20.87	0.0000049	0.00938
DAAM1: + :14:59340073:59355333	− 3.36	3.84	20.40	0.0000063	0.00938
TMCO3: + :13:113520615:113539507	− 3.22	3.85	18.88	0.0000139	0.01662

For each circRNAs following information were provided: gene name, strand, chromosome and genomic position

*logFC* fold-change of the expression in log2 scale, *logCPM* counts per million in log2 scale, *LR* Likelihood Ratio test

p-value and FDR (false discovery rate)

**Fig. 2:**
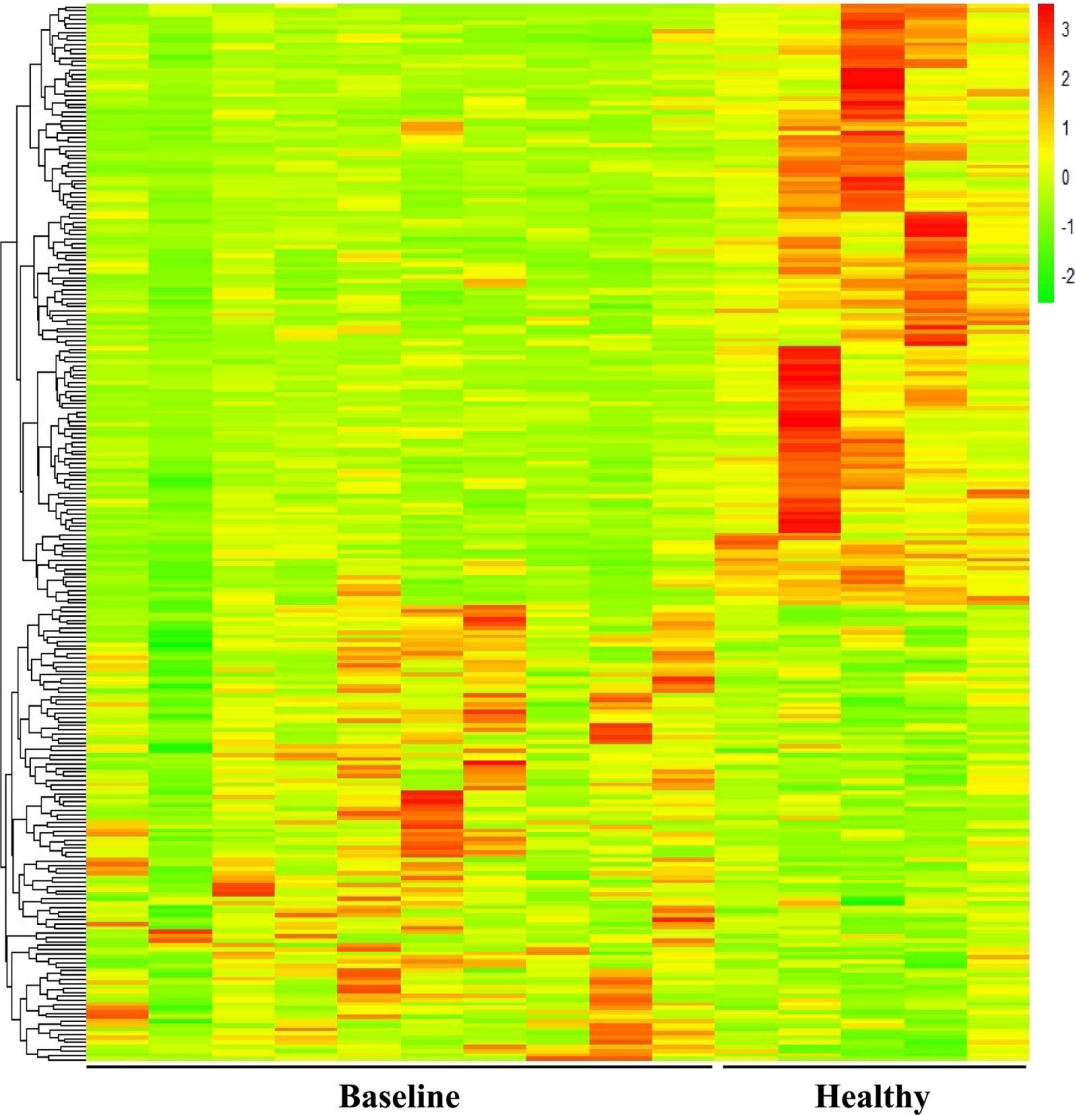
Heatmap showing hierarchical clustering of differentially expressed circRNAs between GEP-NET patients at baseline (n = 10) and healthy donors (n = 5) with p < 0.05. The expression of circRNAs is hierarchically clustered on the y-axis. Colour key indicates the Z score-converted expression values: dark green = lowest, dark red = highest

To better examine the expression of circRNAs in GEP-NET patients compared to controls we performed a hierarchical clustering analysis highlighting the segregation of circRNAs between the compared groups with different expression patterns (Fig. 2).

Hierarchical clustering showed that circRNA expression patterns among samples were distinguishable with an overall down-regulation of circRNA in NET samples compared to controls.

### Changes in GEP-NET circRNA signature at early follow-up after treatment

To explore the potential early predictive role of circRNAs in NETs, we compared circRNAs expression profile in a subgroup of 5 NET patients at baseline and after 3 months from somatostatin analog (SSA) therapy (n = 4) or surgery (n = 1), as shown in Table 2. The patients didn’t show a significant difference in platelets count at baseline and follow-up (p = 0.465) An average of 9269 circRNAs were detected to be differentially expressed with p ≤ 0.05 in all follow-up examined samples. Among them, 4856 and 4771 circRNAs were upregulated and downregulated, respectively. Filtering analysis with p ≤ 0.05 and FDR ≤ 0.05 we identified an average of 4983 remarkably differentially expressed circRNAs between follow-up and baseline samples, among them 2648 up-regulated and 2334 down-regulated. Notably applying p ≤ 0.05 and FDR ≤ 0.05 filters, only 3/5 comparisons gave statistically significant results (Table 2).

**Fig. 3:**
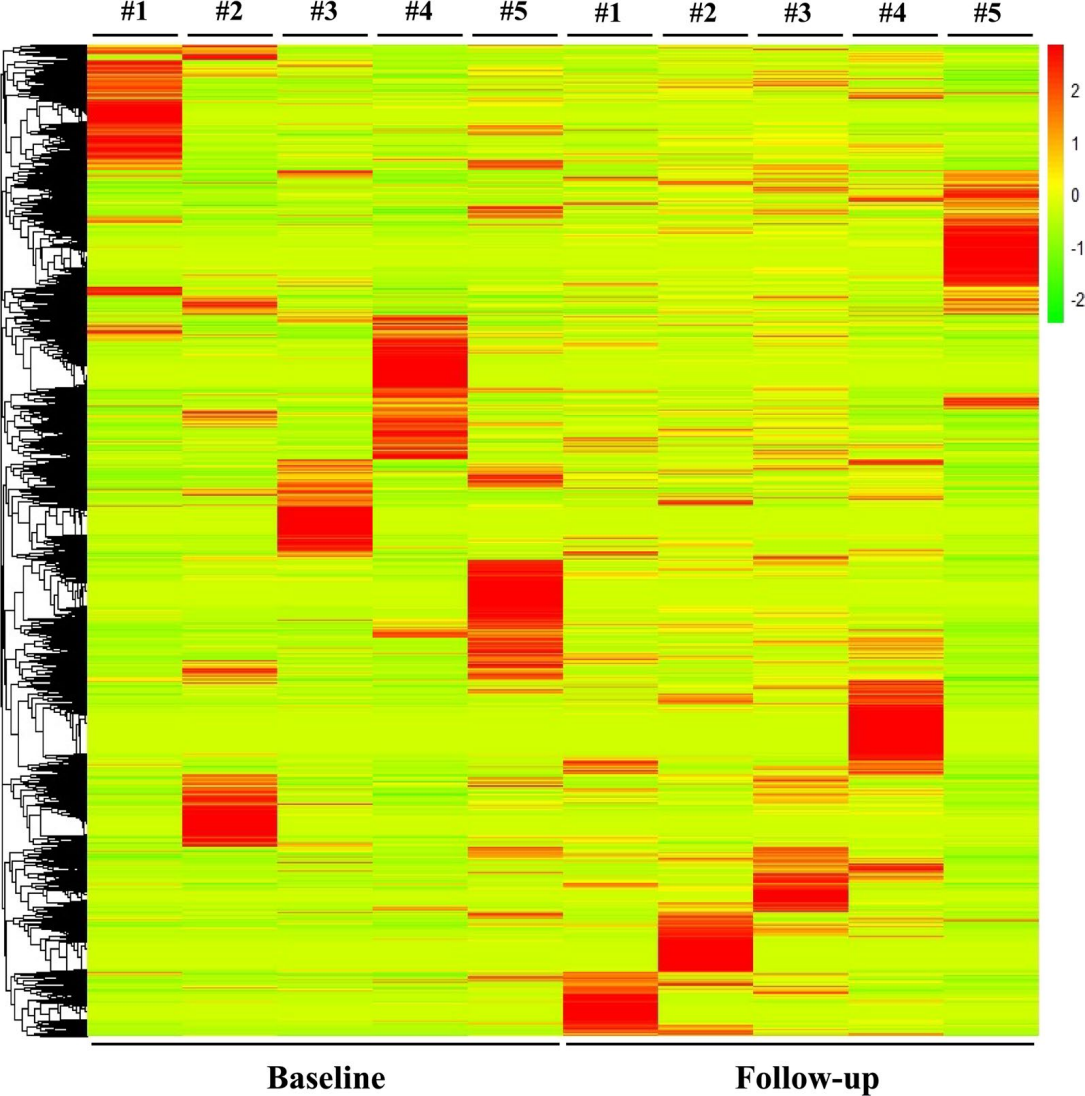
Heatmap showing hierarchical clustering of differentially expressed circRNAs between Baseline and Follow-up GEP-NET patients (n = 5) with p < 0.05. The expression of circRNAs is hierarchically clustered on the y-axis. Colour key indicates the Z score-converted expression values: dark green = lowest, dark red = highest

Hierarchical clustering analysis of circRNAs based on p ≤ 0.05, shows a different expression pattern between follow-up and baseline samples (Fig. 3) for all comparisons, highlighting the potential of treatment to modify the circRNA profile in GEP-NET patients.

**Fig. 4:**
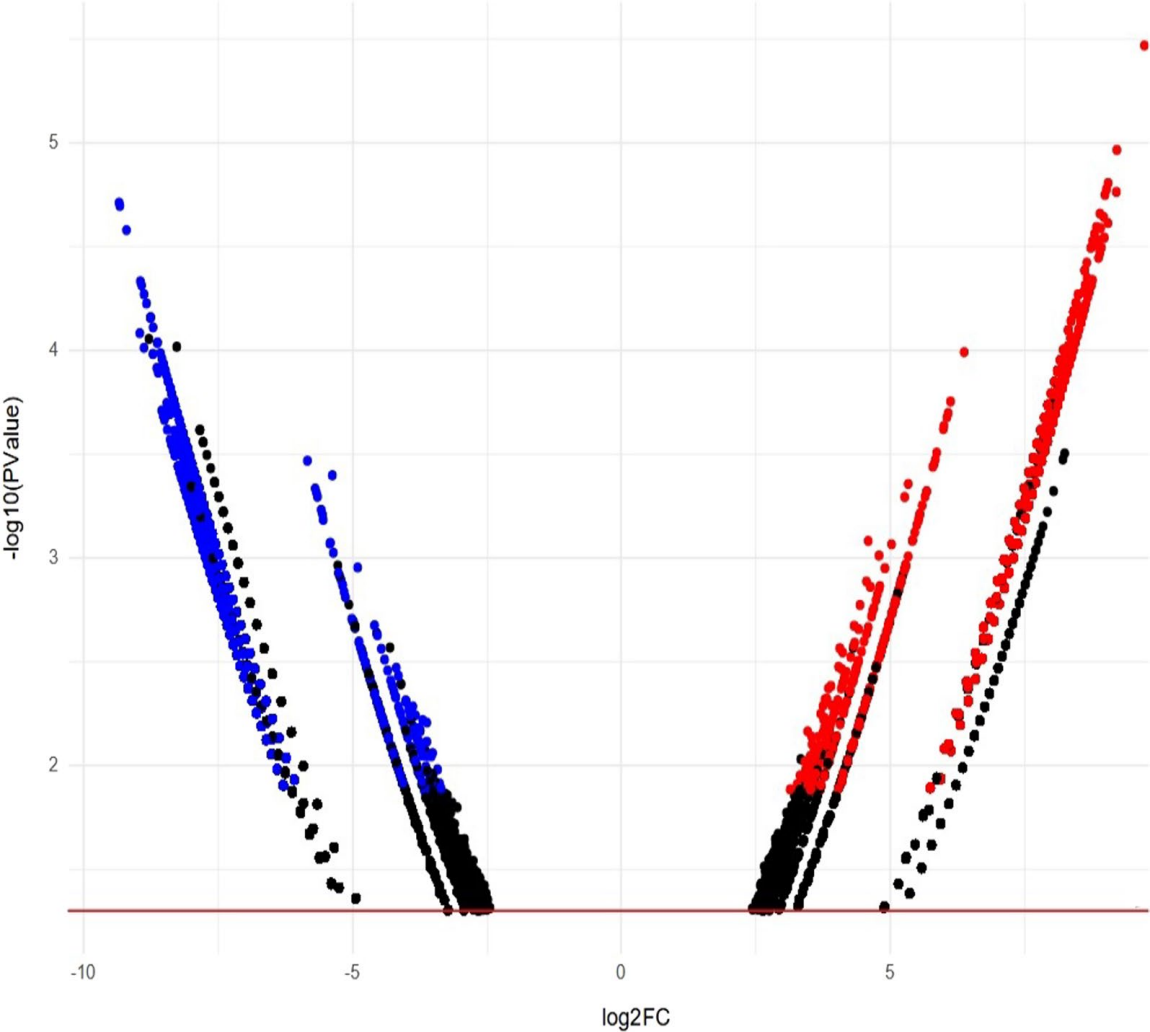
Volcano plot of differentially expressed circRNAs (Follow-up GEP-NET patients *vs* Baseline GEP-NET patients, n = 5). The negative log10 of the p-value is plotted on the y-axis, and the log2 of the FC is plotted on the x-axis. Red line marks p = 0.05. Labelled red and blue dots indicate upregulated and downregulated circRNAs respectively, with p < 0.05 and FDR < 0.05

This is further confirmed applying a more restrictive statistical filter as appreciable in Fig. 4. Differentially expressed circRNAs in follow-up *vs* baseline comparison were used to perform a GO pathways functional enrichment analysis (Additional file 1: Figures S2–S7). This analysis revealed that the overlapping upregulated mRNAs with an enrichment score > 3 were mainly associated with the following terms: chromatin organization, cell cycle and cell division in the Biological Process (BP) category (Additional file 1: Figures S2–S4, top); chromatin binding, GTPase activator activity and ATPase activity in the Molecular Function (MF) category (Additional file 1: Figures S2–S4, middle); centrosome and microtubule organization in the Cellular Component (CC) category (Additional file 1: Figures S2–S4, bottom). The analysis of overlapping down regulated mRNAs with an enrichment score > 3 revealed that they are mainly associated with the following terms: chromatin organization and cell division in the BP category (Additional file 1: Figures S5–S7, top); ATPase activity and helicase activity in the MF category (Additional file 1: Figures S5–S7, middle); centriole and spindle in the CC (Additional file 1: Figures S5–S7, bottom).

## Discussion

The present proof-of-concept study shows, for the first time, to the best of our knowledge, the analysis of circRNA from tumor-educated platelets in a homogeneous sample of G1-G2 GEP-NETs, giving reason to interpret circRNAs as potential new biomarkers in this research field. The use of liquid biopsy in oncology is receiving growing attention, both for an early diagnosis and for tailoring treatment strategies and predicting drug resistance. However, its application in the NEN field is limited to NETest, which has been demonstrated to have not only a diagnostic role but also a prognostic and predictive role [24]. The term liquid biopsy encloses the research of nucleic acids (RNA and DNA), circulating tumor cells, exosomes, and TEPs [18]. TEPs and their circRNA cargo were never analyzed in NENs.

The interplay between platelets and tumor cells is involved in tumor growth and dissemination [25] and platelets can take up tumor-derived secreted membrane vesicles containing RNAs, becoming TEPs [14]. It is therefore possible to isolate platelets to have access to tumor RNA, a potential biomarker for cancer diagnostics. CircRNA is a ncRNA, originating from back splicing, characterized by a covalently closed loop structure due to the bind of 5’ splice site with upstream 3’ splicing site of a pre-mRNA molecule, gaining stability. CircRNAs play many roles: inhibitors of microRNA or protein (acting as ‘sponges’), regulators of protein function through the binding of specific proteins to multiple circRNAs (acting as a molecular reservoir of proteins), and more rarely, being translated (coding circRNAs) [26]. In non-neuroendocrine tumors, the evaluation of circRNAs from TEPs can discriminate patients affected by neoplasms from healthy subjects and gives the possibility to identify the primary tumor histotype and detect possible predictors of treatment response [16]. This approach has been used in many kinds of cancers, including non-small cell lung cancer [27], colorectal cancer [28], glioblastoma [28], renal cell carcinoma [29], sarcoma [30], prostate cancer [31], and hepatocellular carcinoma [32].

We sequenced up to one hundred thousand circRNAs expressed in NET patients and controls, with most of them not previously annotated in databases [22]. This data confirmed the potential validity of this strategy also in GEP-NETs, differently from other sources of liquid biopsy, such as ctDNA, which are less released in the case of well-differentiated NENs [33].

Our study demonstrated that 5 circRNAs are differentially expressed in patients with NETs compared to controls. These genes are known to be involved in the initiation and progression of different cancer types (Table 4).

**Table 4:** Expression pattern of circRNAs significantly differentially expressed in GEP-NET patients compared to controls

Gene	Tumor	Expression pattern	Ref.
USP32	Ovarian cancer	Overexpressed	[34]
	Gastric cancer	Overexpressed	[35]
	Glioblastoma	Overexpressed	[36]
	Breast cancer	Overexpressed	[37]
	Small cell lung cancer	Overexpressed	[38]
	Pancreatic ductal adenocarcinoma	Overexpressed	[39]
IQGAP2	Gastric cancer	Downregulated	[43]
	Prostate cancer	Downregulated	[44]
	Hepatocellular carcinoma	Downregulated	[45]
	Ovarian cancer	Downregulated	[46]
DAAM1	Breast cancer	Overexpressed	[48]
	Glioblastoma	Overexpressed	[49]
	Ovarian cancer	Overexpressed	[50]
TMCO3	Hepatocellular carcinoma	Overexpressed	[51, 52]
	Neuroblastoma	Overexpressed	[53]

*USP32* belongs to the ubiquitin-specific protease family, deubiquitinating enzymes which have been reported to be involved in several cancer initiation and progression (ovarian cancer [34], gastric cancer [35], glioblastoma [36], breast cancer [37], small cell lung cancer [38]). A recent work suggests a pivotal role of *USP32* in pancreatic ductal adenocarcinoma given its higher expression levels compared to normal pancreatic tissues and a significant association with tumor grade and stage [39].

*EXOC5* is a central component of the exocyst complex essential for targeting exocytic vesicles to specific docking sites on the plasma membrane. It is required for photoreceptor ciliogenesis and retinal development [40]. A recent work demonstrated that circEXOC5 (hsa_circ_0004399) is one of the mostly significantly up regulated circRNAs identified by microarray in lung macrophages from acute lung injured mice compared to control mice finally demonstrating a pivotal role of this circRNAs in promoting acute lung injury in mice [41].

*IQGAP2* belongs to the scaffold protein family of IQ motif-containing GTPase-activating proteins [42]. *IQGAP2* expression is reduced and plays a tumor suppressor role in most solid cancer types wherein reduced levels of *IQGAP2* correlated with poor overall survival of patients (gastric cancer [43], prostate cancer [44], hepatocellular carcinoma [45], ovarian cancer [46]).

*DAAM1* is a formin protein involved in cytoskeletal rearrangement and phagocytosis [47]. Many works demonstrated a dysregulated expression of *DAAM1* in different tumors. Among them Mei et al. have found that *DAMM1* overexpression correlates with metastasis and predicts poor prognosis in breast cancer [48] while others found *DAMM1* overexpression in other tumors such as glioblastoma [49] and ovarian cancer [50].

*TMCO3* is a member of the proton transducer 2 family of transporter proteins. A recent work demonstrated a significant overexpression of *TMCO3* and a pivotal role in tumor progression in liver hepatocellular carcinoma [51, 52]. A strong correlation between *TMCO3* and cancer also comes from a whole genome sequencing analysis on cfDNA from neuroblastoma patients in which *TMCO3* was found highly mutated compared to healthy subjects [53].

These 5 potential biomarkers are ideal candidates for further validation by reverse transcription–quantitative PCR (RT-qPCR).

To assess the function of differentially expressed circRNAs we performed a GO pathway enrichment analysis. This analysis revealed that highly significant DE-genes are mainly involved in the regulation of cell cycle and cell division. Signaling pathways involved in the regulation of cell cycle play a pivotal role in driving the growth and development of several types of cancer including NETs [54, 55].

The study demonstrated also that treatment with either surgery or SSA can modulate circRNAs expression. An average of 9269 circRNAs (4856 upregulated and 4771 downregulated) were detected to be differentially expressed in all follow-up examined samples. Using double filters, we identified an average of 4983 (2648 up-regulated and 2334 down-regulated) remarkably differentially expressed circRNAs between follow-up and baseline samples. Notably applying p ≤ 0.05 and FDR ≤ 0.05 filters, only 3/5 comparisons gave statistically significant results. Among these 3 patients one is a woman affected by a functioning metastatic PNET (insulinoma), she underwent surgical treatment with an excellent response after surgery and was disease-free at last follow-up. The other two patients are men affected by metastatic NET (one originating from pancreas/duodenum, one from ileum), who started medical treatment with SSA. Both of them showed a partial response in terms of reduction of metastatic lesions. GO analysis reveals that in these 3 patients the most enriched biological pathways involve cell cycle, cell division, chromatin organization, and DNA damage repair. However, these data must be considered preliminary and need to be confirmed on a larger sample of patients. The two other patients who were followed-up after medical treatment without statistically significant changes in circRNAs expression showed stable disease at the time of the second blood collection, suggesting that an improved clinical course could be associated to statistically significant circRNAs changes.

## Conclusions

The present proof-of-concept study allows to: (i) define for the first time circRNAs signature from TEPs in GEP-NETs demonstrating that it could be a viable diagnostic biomarker in these patients; (ii) identify 5 circRNAs differentially expressed in NET patients compared to controls; (iii) show a significantly different circRNAs expression at early follow up after standard treatment in a subgroup of patients exhibiting complete or partial response after treatment, suggesting also a potential early predictive role of TEPs-derived circRNAs.

## Limitations

The main limitation of this study is the small sample size. However, we are continuing to enroll patients for this project to further confirm and validate these data. Further analyses will be necessary to verify the reliability of the RNA-seq data. These analyses will require a dedicated work in which most differentially expressed circRNAs candidates will be validated by Real Time quantitative Polymerase Chain Reaction (RTqPCR) in a larger sample cohort.

### Supplementary Information

**Additional file 1: Figure S1.** Structural and genomic features of circRNAs. **Figure S2.** Tree map of the enriched GO category (Biological Process, Molecular Function and Cellular Component) among the up-regulated genes for Follow up *vs* Baseline GEP-NET#1 comparison. **Figure S3.** Tree map of the enriched GO category (Biological Process, Molecular Function and Cellular Component) among the up-regulated genes for Follow up *vs* Baseline GEP-NET#4 comparison. **Figure S4.** Tree map of the enriched GO category (Biological Process, Molecular Function and Cellular Component) among the up-regulated genes for Follow up *vs* Baseline GEP-NET#5 comparison. **Figure S5.** Tree map of the enriched GO category (Biological Process, Molecular Function and Cellular Component) among the down-regulated genes for Follow up *vs* Baseline GEP-NET#1 comparison. **Figure S6.** Tree map of the enriched GO category (Biological Process, Molecular Function and Cellular Component) among the down-regulated genes for Follow up *vs* Baseline GEP-NET#4 comparison. **Figure S7.** Tree map of the enriched GO category (Biological Process, Molecular Function and Cellular Component) among the down-regulated genes for Follow up *vs* Baseline GEP-NET#5 comparison. **Table S1.** List of all circRNAs identified in the whole cohort and relative annotations.

## Data Availability

The datasets used and/or analyzed during the current study are available from the corresponding author on reasonable request.

## Abbreviations

NENs: Neuroendocrine neoplasms
NETs: Neuroendocrine tumors
NECs: Neuroendocrine carcinomas
circRNAs: Circular RNAs
ncRNAs: Non-coding RNAs
miRNAs: MicroRNA
TEPs: Tumor-educated platelets
GEP: Gastroenteropancreatic
GO: Gene ontology
SSA: Somatostatin analog
BP: Biological process
MF: Molecular function
CC: Cellular component
