# Supplementary material for: Platelet-derived circRNAs signature in patients with gastroenteropancreatic neuroendocrine tumors
Source: J Transl Med. 2023 Aug 16;21:548. doi: 10.1186/s12967-023-04417-8 (PMC10428534; doi:10.1186/s12967-023-04417-8)
Supplement: Supplementary file 1 — Additional file 1: Figure S1. Structural and genomic features of circRNAs. Figure S2. Tree map of the enriched GO category (Biological Process, Molecular Function and Cellular Component) among the up-regulated genes for Follow up vs Baseline GEP-NET#1 comparison. Figure S3. Tree map of the enriched GO category (Biological Process, Molecular Function and Cellular Component) among the up-regulated genes for Follow up vs Baseline GEP-NET#4 comparison. Figure S4. Tree map of the enriched GO category (Biological Process, Molecular Function and Cellular Component) among the up-regulated genes for Follow up vs Baseline GEP-NET#5 comparison. Figure S5. Tree map of the enriched GO category (Biological Process, Molecular Function and Cellular Component) among the down-regulated genes for Follow up vs Baseline GEP-NET#1 comparison. Figure S6. Tree map of the enriched GO category (Biological Process, Molecular Function and Cellular Component) among the down-regulated genes for Follow up vs Baseline GEP-NET#4 comparison. Figure S7. Tree map of the enriched GO category (Biological Process, Molecular Function and Cellular Component) among the down-regulated genes for Follow up vs Baseline GEP-NET#5 comparison. Table S1. List of all circRNAs identified in the whole cohort and relative annotations. [file 12967_2023_4417_MOESM1_ESM.zip › Additional file/Figure S2.pdf]

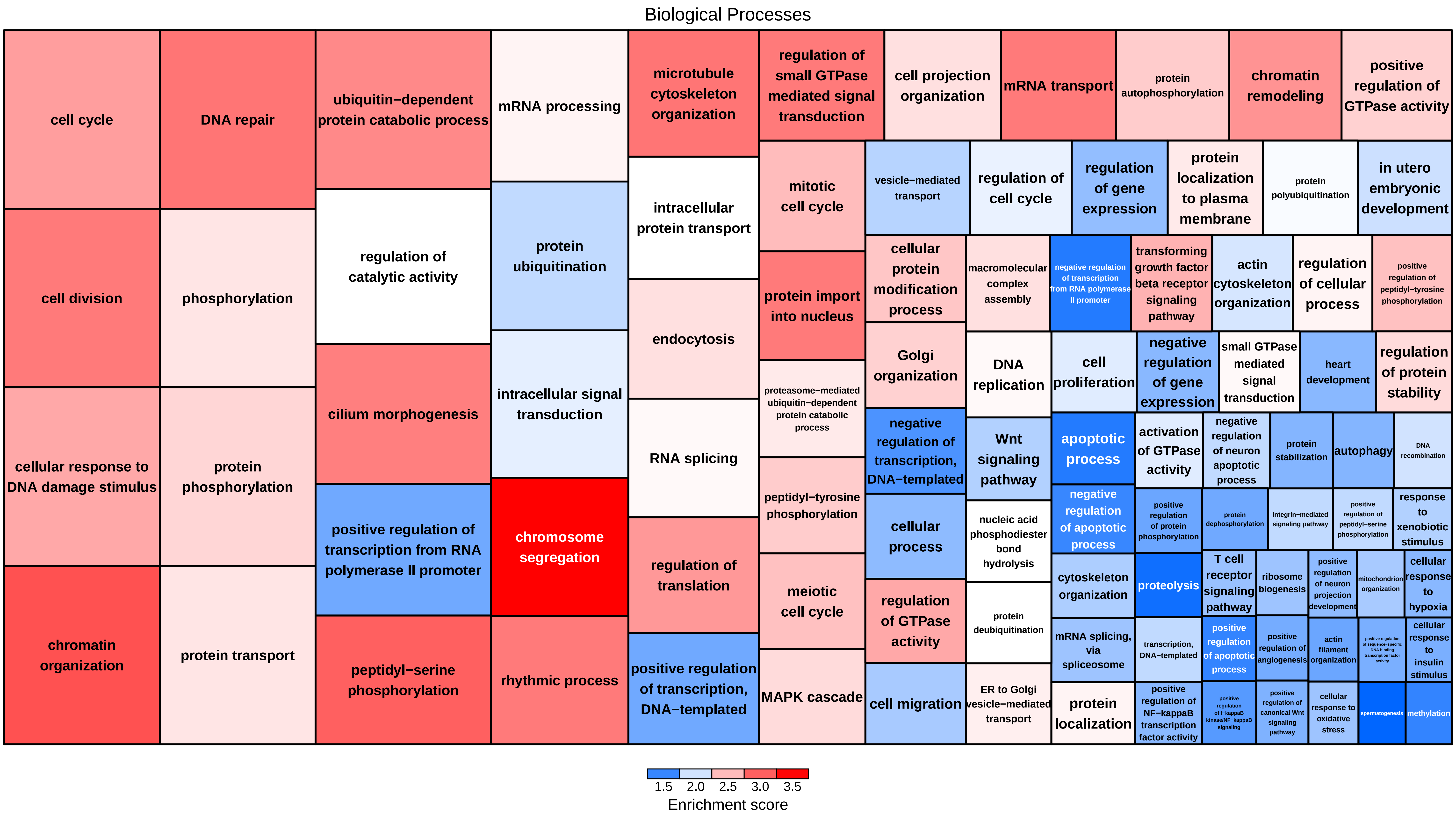

Biological Processes
cell cycle
DNA repair
ubiquitin−dependent protein catabolic process
mRNA processing
microtubule cytoskeleton organization
regulation of small GTPase mediated signal transduction
cell projection organization
mRNA transport
protein autophosphorylation
chromatin remodeling
positive regulation of GTPase activity
intracellular protein transport
mitotic cell cycle
vesicle−mediated transport
regulation of cell cycle
regulation of gene expression
protein localization to plasma membrane
protein polyubiquitination
in utero embryonic development
regulation of catalytic activity
protein ubiquitination
cellular protein modification process
macromolecular complex assembly
negative regulation of transcription from RNA polymerase II promoter
transforming growth factor beta receptor signaling pathway
actin cytoskeleton organization
regulation of cellular process
positive regulation of peptidyl−tyrosine phosphorylation
cell division
phosphorylation
endocytosis
protein import into nucleus
Golgi organization
DNA replication
cell proliferation
negative regulation of gene expression
small GTPase mediated signal transduction
heart development
regulation of protein stability
cilium morphogenesis
intracellular signal transduction
proteasome−mediated ubiquitin−dependent protein catabolic process
negative regulation of transcription, DNA−templated
Wnt signaling pathway
apoptotic process
activation of GTPase activity
negative regulation of neuron apoptotic process
protein stabilization
autophagy
DNA recombination
cellular response to DNA damage stimulus
protein phosphorylation
RNA splicing
peptidyl−tyrosine phosphorylation
cellular process
nucleic acid phosphodiester bond hydrolysis
negative regulation of apoptotic process
positive regulation of protein phosphorylation
protein dephosphorylation
integrin−mediated signaling pathway
positive regulation of peptidyl−serine phosphorylation
response to xenobiotic stimulus
positive regulation of transcription from RNA polymerase II promoter
chromosome segregation
regulation of translation
meiotic cell cycle
regulation of GTPase activity
protein deubiquitination
cytoskeleton organization
proteolysis
T cell receptor signaling pathway
ribosome biogenesis
positive regulation of neuron projection development
mitochondrion organization
cellular response to hypoxia
mRNA splicing, via spliceosome
transcription, DNA−templated
positive regulation of apoptotic process
positive regulation of angiogenesis
actin filament organization
positive regulation of sequence−specific DNA binding transcription factor activity
cellular response to insulin stimulus
chromatin organization
protein transport
peptidyl−serine phosphorylation
rhythmic process
positive regulation of transcription, DNA−templated
MAPK cascade
cell migration
ER to Golgi vesicle−mediated transport
protein localization
positive regulation of NF−kappaB transcription factor activity
positive regulation of I−kappaB kinase/NF−kappaB signaling
positive regulation of canonical Wnt signaling pathway
cellular response to oxidative stress
spermatogenesis
methylation
1.5
2.0
2.5
3.0
3.5
Enrichment score

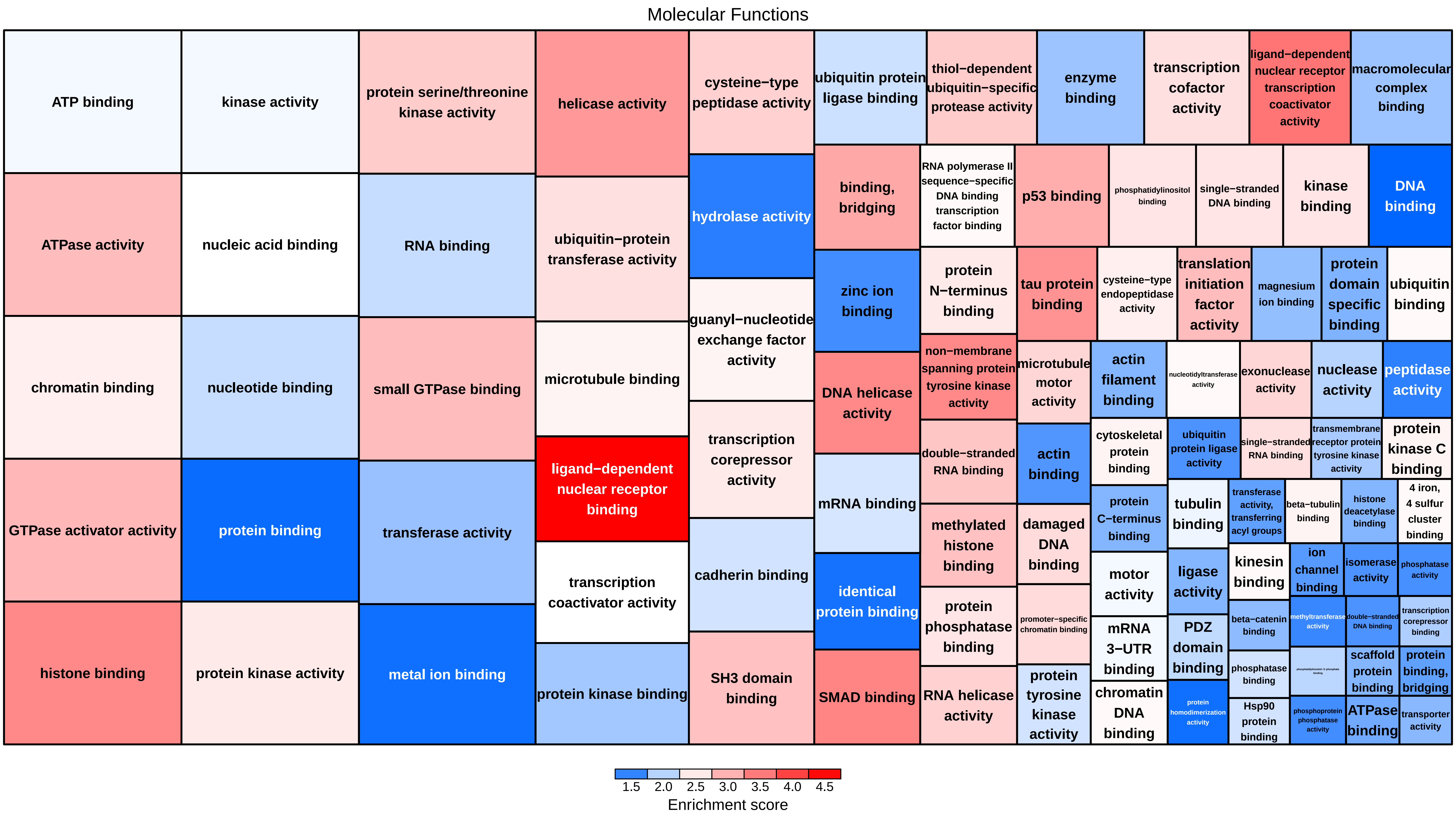
Molecular Functions
ATP binding
ATPase activity
chromatin binding
GTPase activator activity
histone binding
kinase activity
nucleic acid binding
nucleotide binding
protein binding
protein kinase activity
protein serine/threonine kinase activity
RNA binding
small GTPase binding
transferase activity
metal ion binding
helicase activity
ubiquitin−protein transferase activity
microtubule binding
ligand−dependent nuclear receptor binding
transcription coactivator activity
protein kinase binding
cysteine−type peptidase activity
hydrolase activity
guanyl−nucleotide exchange factor activity
transcription corepressor activity
cadherin binding
SH3 domain binding
ubiquitin protein ligase binding
binding, bridging
zinc ion binding
DNA helicase activity
mRNA binding
identical protein binding
SMAD binding
thiol−dependent ubiquitin−specific protease activity
RNA polymerase II sequence−specific DNA binding transcription factor binding
protein N−terminus binding
non−membrane spanning protein tyrosine kinase activity
double−stranded RNA binding
methylated histone binding
protein phosphatase binding
RNA helicase activity
enzyme binding
p53 binding
tau protein binding
microtubule motor activity
actin binding
damaged DNA binding
promoter−specific chromatin binding
protein tyrosine kinase activity
phosphatidylinositol binding
cysteine−type endopeptidase activity
actin filament binding
cytoskeletal protein binding
protein C−terminus binding
motor activity
mRNA 3−UTR binding
chromatin DNA binding
transcription cofactor activity
single−stranded DNA binding
translation initiation factor activity
nucleotidyltransferase activity
ubiquitin protein ligase activity
tubulin binding
ligase activity
PDZ domain binding
protein homodimerization activity
ligand−dependent nuclear receptor transcription coactivator activity
kinase binding
magnesium ion binding
exonuclease activity
single−stranded RNA binding
transferase activity, transferring acyl groups
beta−tubulin binding
kinesin binding
ion channel binding
beta−catenin binding
methyltransferase activity
phosphatase binding
phosphatidylinositol−3−phosphate binding
Hsp90 protein binding
phosphoprotein phosphatase activity
macromolecular complex binding
DNA binding
protein domain specific binding
ubiquitin binding
nuclease activity
peptidase activity
transmembrane receptor protein tyrosine kinase activity
protein kinase C binding
histone deacetylase binding
4 iron, 4 sulfur cluster binding
isomerase activity
phosphatase activity
double−stranded DNA binding
transcription corepressor binding
scaffold protein binding
protein binding, bridging
ATPase binding
transporter activity
1.5
2.0
2.5
3.0
3.5
4.0
4.5
Enrichment score

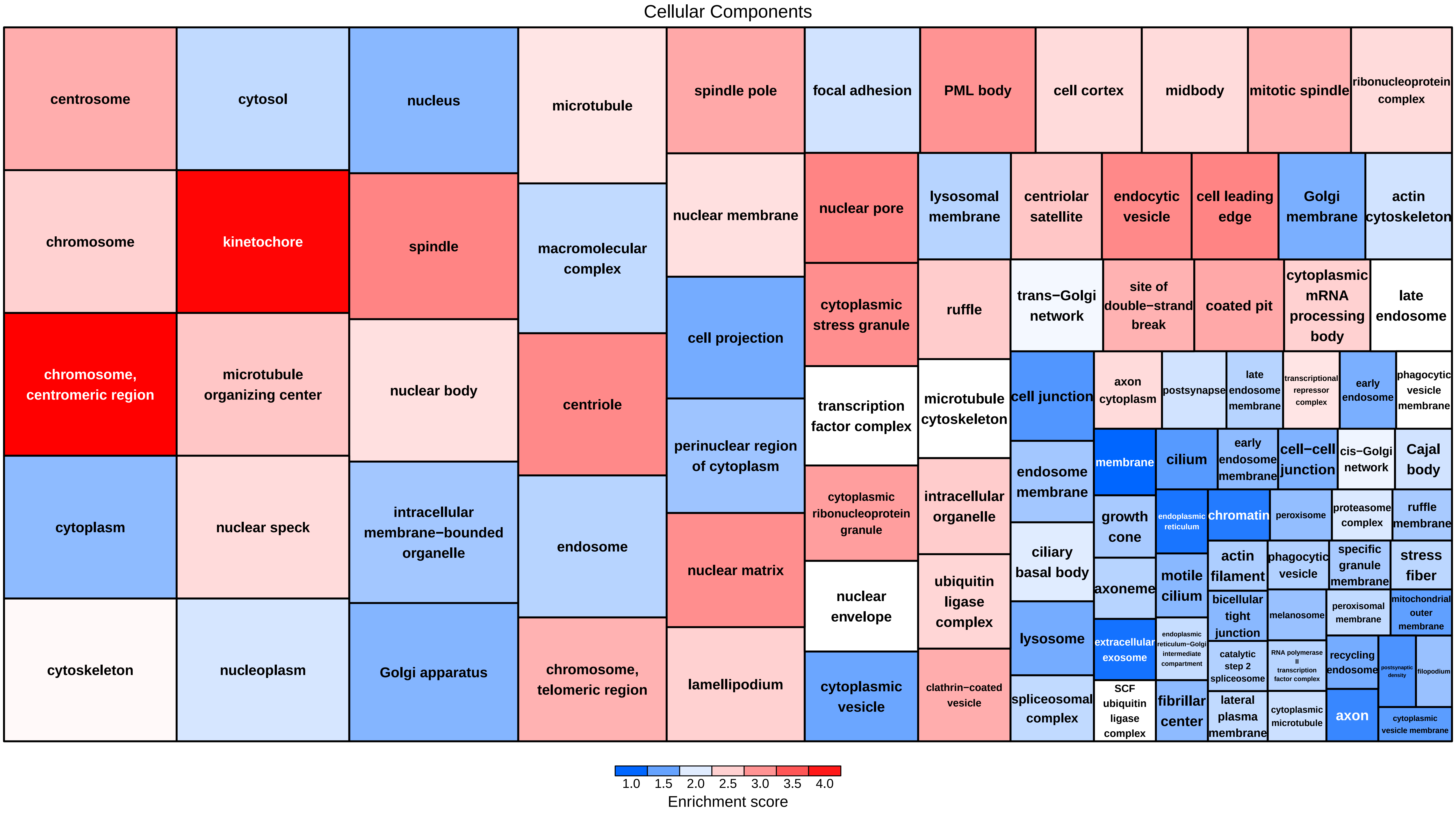
Cellular Components
centrosome
cytosol
nucleus
microtubule
spindle pole
focal adhesion
PML body
cell cortex
midbody
mitotic spindle
ribonucleoprotein complex
chromosome
kinetochore
spindle
macromolecular complex
nuclear membrane
nuclear pore
lysosomal membrane
centriolar satellite
endocytic vesicle
cell leading edge
Golgi membrane
actin cytoskeleton
chromosome, centromeric region
microtubule organizing center
nuclear body
centriole
cell projection
cytoplasmic stress granule
ruffle
trans−Golgi network
site of double−strand break
coated pit
cytoplasmic mRNA processing body
late endosome
transcription factor complex
microtubule cytoskeleton
cell junction
axon cytoplasm
postsynapse
late endosome membrane
transcriptional repressor complex
early endosome
phagocytic vesicle membrane
perinuclear region of cytoplasm
membrane
cilium
early endosome membrane
cell−cell junction
cis−Golgi network
Cajal body
cytoplasm
nuclear speck
intracellular membrane−bounded organelle
endosome
cytoplasmic ribonucleoprotein granule
intracellular organelle
endosome membrane
growth cone
endoplasmic reticulum
chromatin
peroxisome
proteasome complex
ruffle membrane
nuclear matrix
nuclear envelope
ubiquitin ligase complex
ciliary basal body
axoneme
motile cilium
actin filament
phagocytic vesicle
specific granule membrane
stress fiber
bicellular tight junction
melanosome
peroxisomal membrane
mitochondrial outer membrane
lysosome
extracellular exosome
endoplasmic reticulum−Golgi intermediate compartment
catalytic step 2 spliceosome
RNA polymerase II transcription factor complex
recycling endosome
postsynaptic density
filopodium
cytoskeleton
nucleoplasm
Golgi apparatus
chromosome, telomeric region
lamellipodium
cytoplasmic vesicle
clathrin−coated vesicle
spliceosomal complex
SCF ubiquitin ligase complex
fibrillar center
lateral plasma membrane
cytoplasmic microtubule
axon
cytoplasmic vesicle membrane
1.0
1.5
2.0
2.5
3.0
3.5
4.0
Enrichment score
