# Supplementary figures and images for: Platelet-derived circRNAs signature in patients with gastroenteropancreatic neuroendocrine tumors
Source: J Transl Med. 2023 Aug 16;21:548. doi: 10.1186/s12967-023-04417-8 (PMC10428534; doi:10.1186/s12967-023-04417-8)

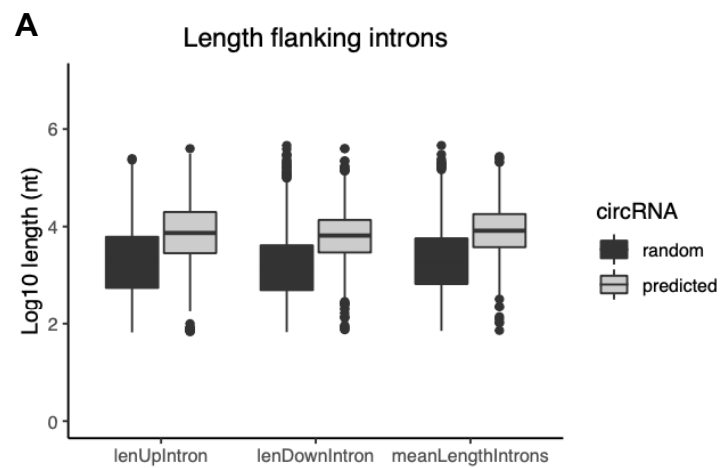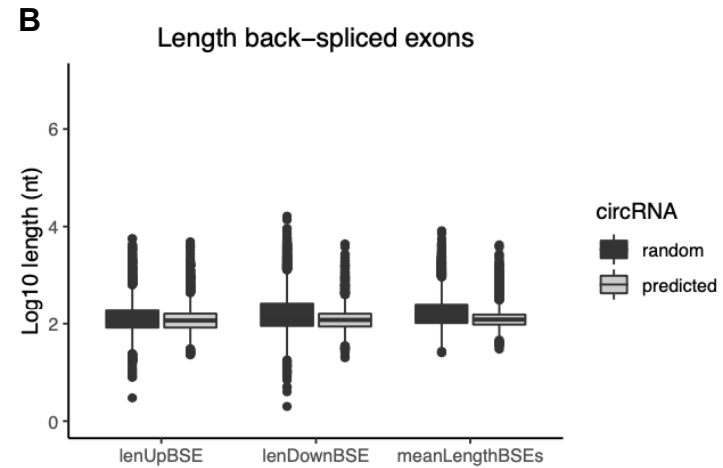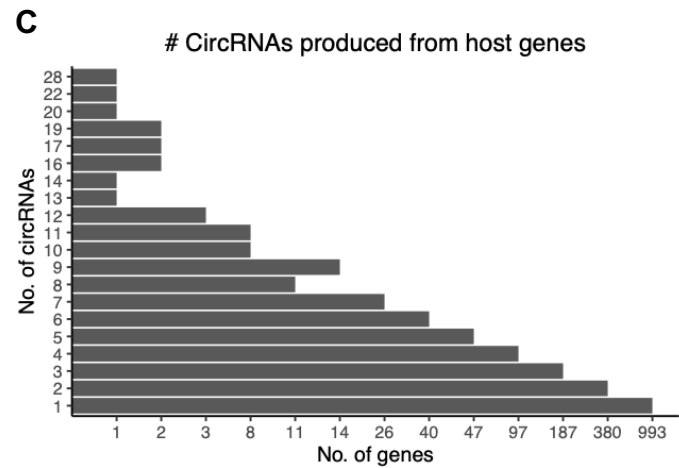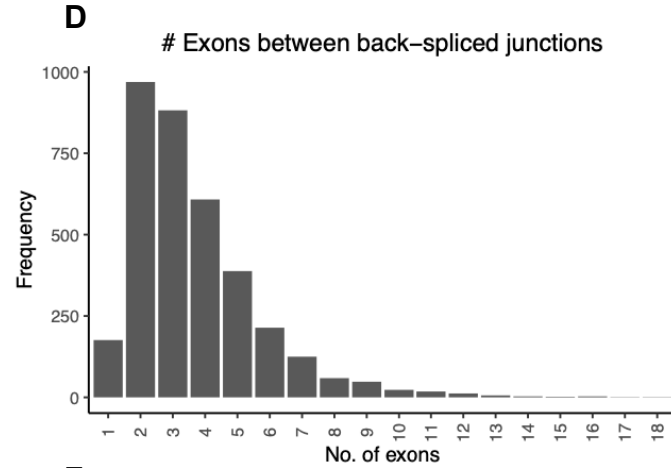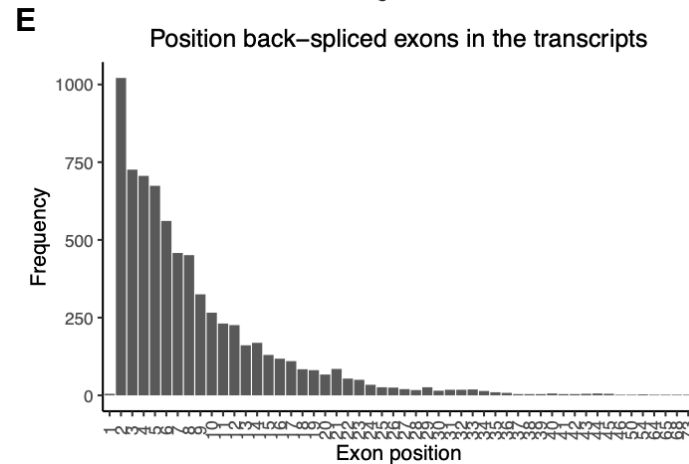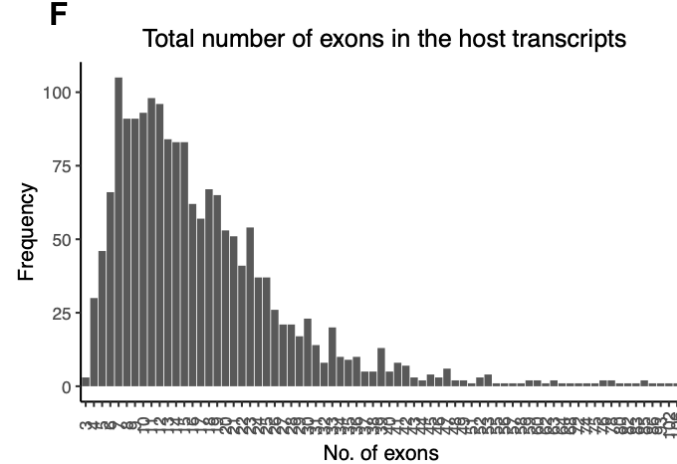

Supplement: Supplementary file 1 — Additional file 1: Figure S1. Structural and genomic features of circRNAs. Figure S2. Tree map of the enriched GO category (Biological Process, Molecular Function and Cellular Component) among the up-regulated genes for Follow up vs Baseline GEP-NET#1 comparison. Figure S3. Tree map of the enriched GO category (Biological Process, Molecular Function and Cellular Component) among the up-regulated genes for Follow up vs Baseline GEP-NET#4 comparison. Figure S4. Tree map of the enriched GO category (Biological Process, Molecular Function and Cellular Component) among the up-regulated genes for Follow up vs Baseline GEP-NET#5 comparison. Figure S5. Tree map of the enriched GO category (Biological Process, Molecular Function and Cellular Component) among the down-regulated genes for Follow up vs Baseline GEP-NET#1 comparison. Figure S6. Tree map of the enriched GO category (Biological Process, Molecular Function and Cellular Component) among the down-regulated genes for Follow up vs Baseline GEP-NET#4 comparison. Figure S7. Tree map of the enriched GO category (Biological Process, Molecular Function and Cellular Component) among the down-regulated genes for Follow up vs Baseline GEP-NET#5 comparison. Table S1. List of all circRNAs identified in the whole cohort and relative annotations. [file 12967_2023_4417_MOESM1_ESM.zip › Additional file/Figure S1.pdf]

Biological Processes

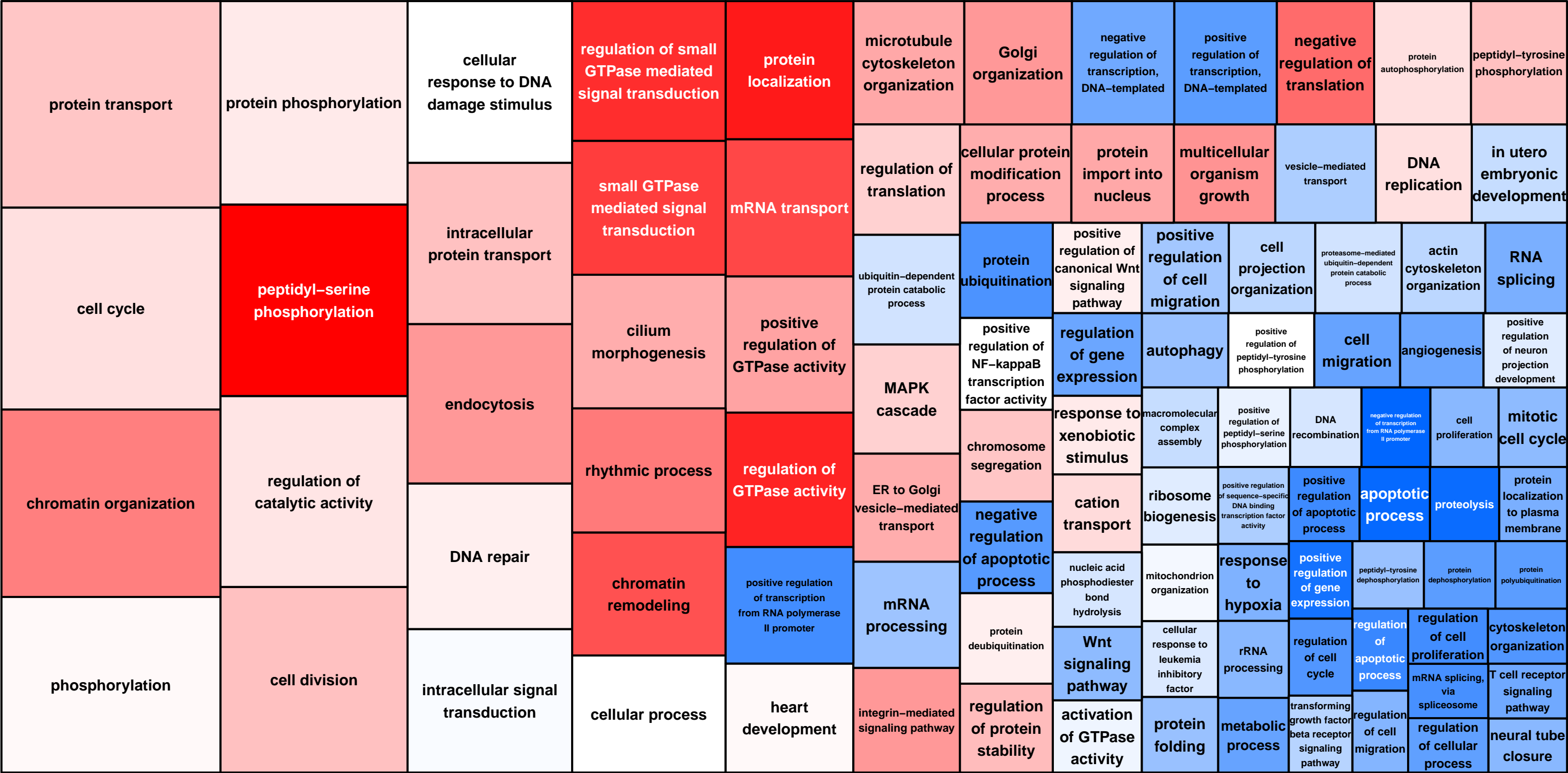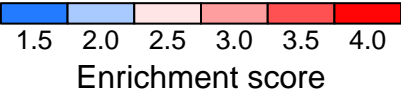

Molecular Functions

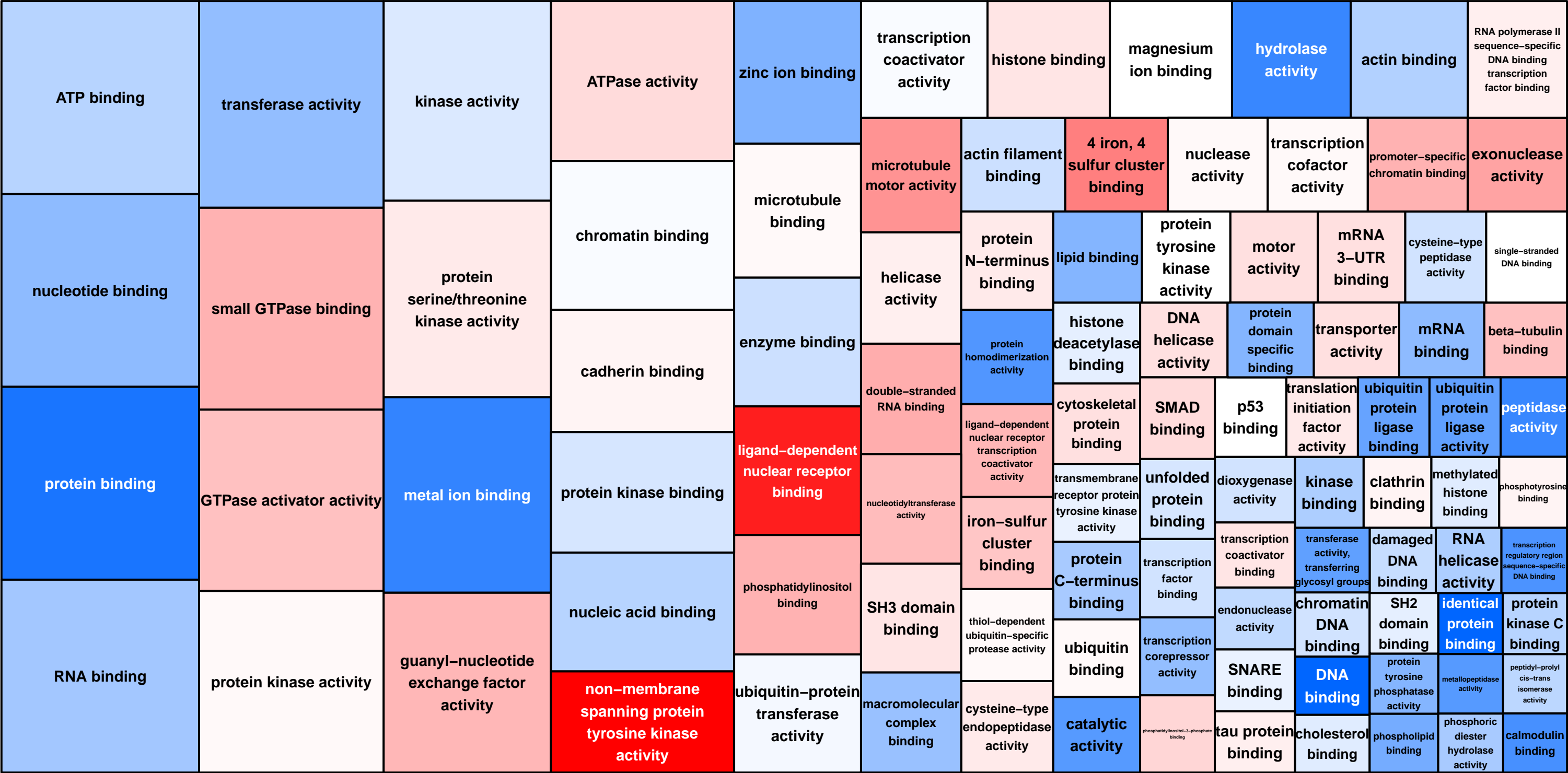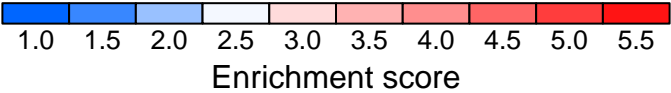

Cellular Components

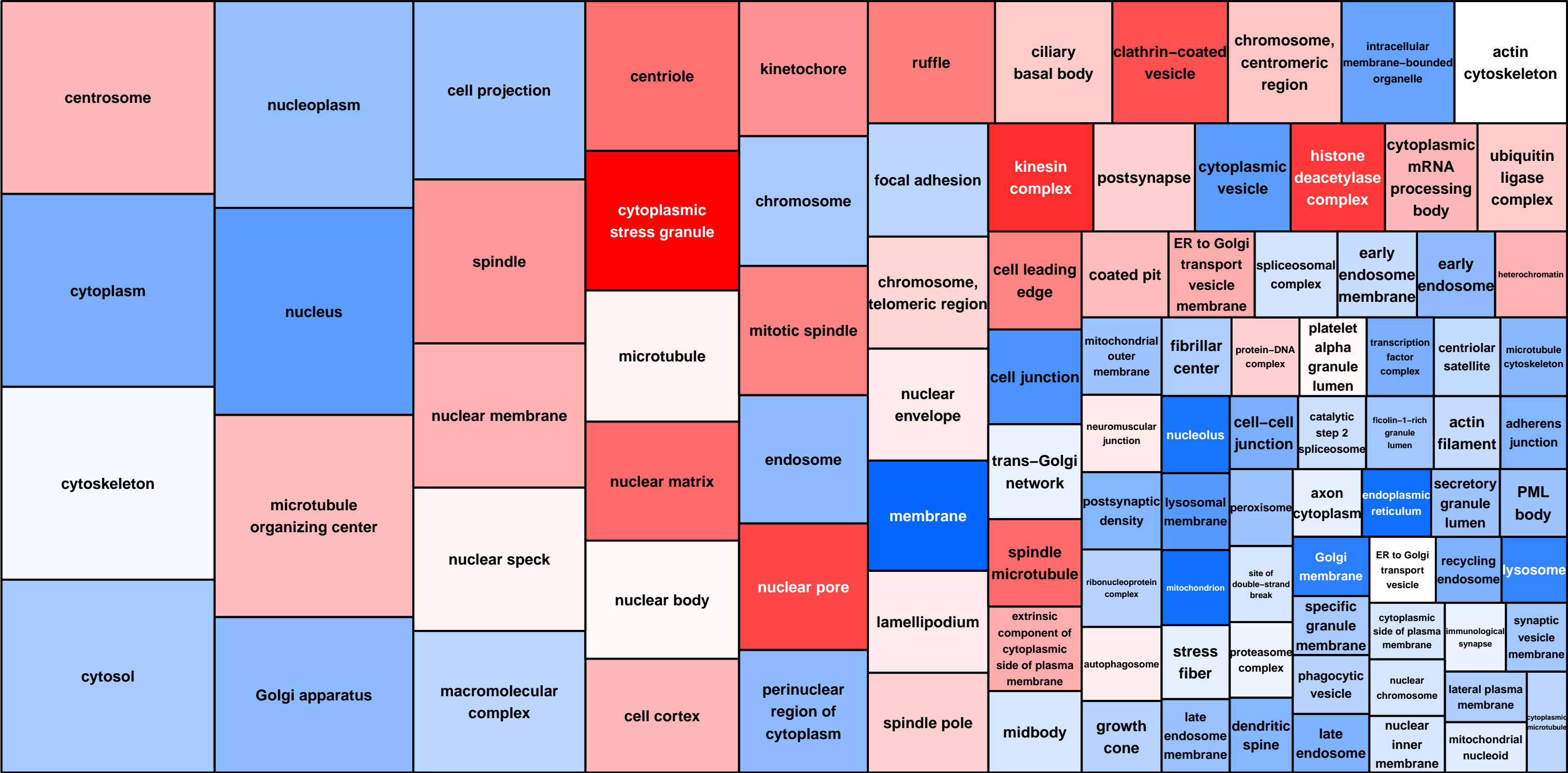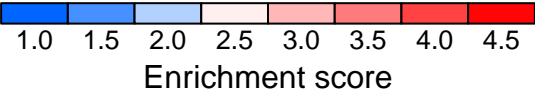

Supplement: Supplementary file 1 — Additional file 1: Figure S1. Structural and genomic features of circRNAs. Figure S2. Tree map of the enriched GO category (Biological Process, Molecular Function and Cellular Component) among the up-regulated genes for Follow up vs Baseline GEP-NET#1 comparison. Figure S3. Tree map of the enriched GO category (Biological Process, Molecular Function and Cellular Component) among the up-regulated genes for Follow up vs Baseline GEP-NET#4 comparison. Figure S4. Tree map of the enriched GO category (Biological Process, Molecular Function and Cellular Component) among the up-regulated genes for Follow up vs Baseline GEP-NET#5 comparison. Figure S5. Tree map of the enriched GO category (Biological Process, Molecular Function and Cellular Component) among the down-regulated genes for Follow up vs Baseline GEP-NET#1 comparison. Figure S6. Tree map of the enriched GO category (Biological Process, Molecular Function and Cellular Component) among the down-regulated genes for Follow up vs Baseline GEP-NET#4 comparison. Figure S7. Tree map of the enriched GO category (Biological Process, Molecular Function and Cellular Component) among the down-regulated genes for Follow up vs Baseline GEP-NET#5 comparison. Table S1. List of all circRNAs identified in the whole cohort and relative annotations. [file 12967_2023_4417_MOESM1_ESM.zip › Additional file/Figure S3.pdf]

Biological Processes

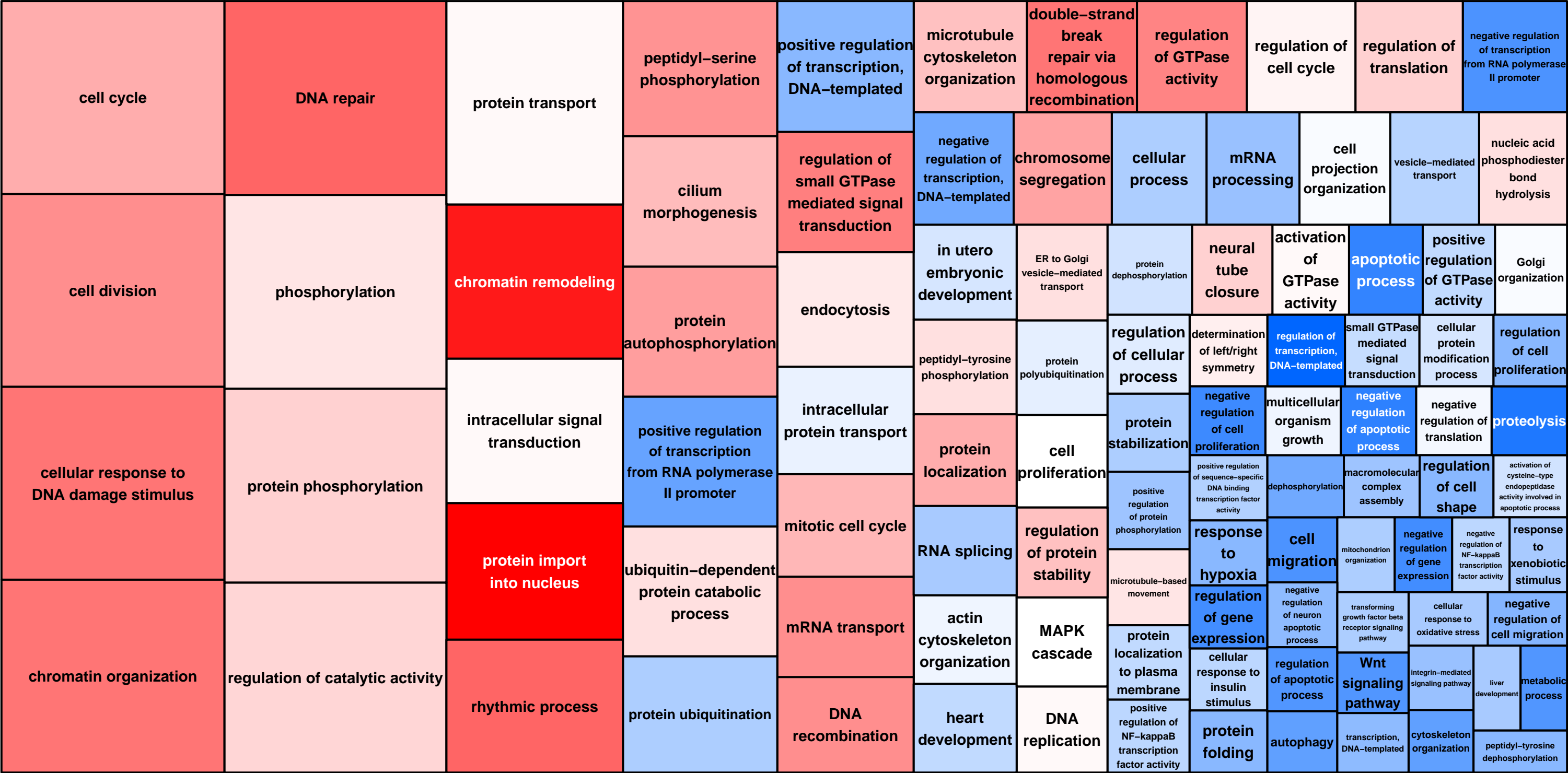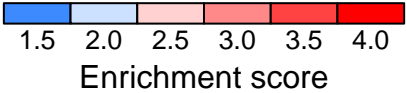

Molecular Functions

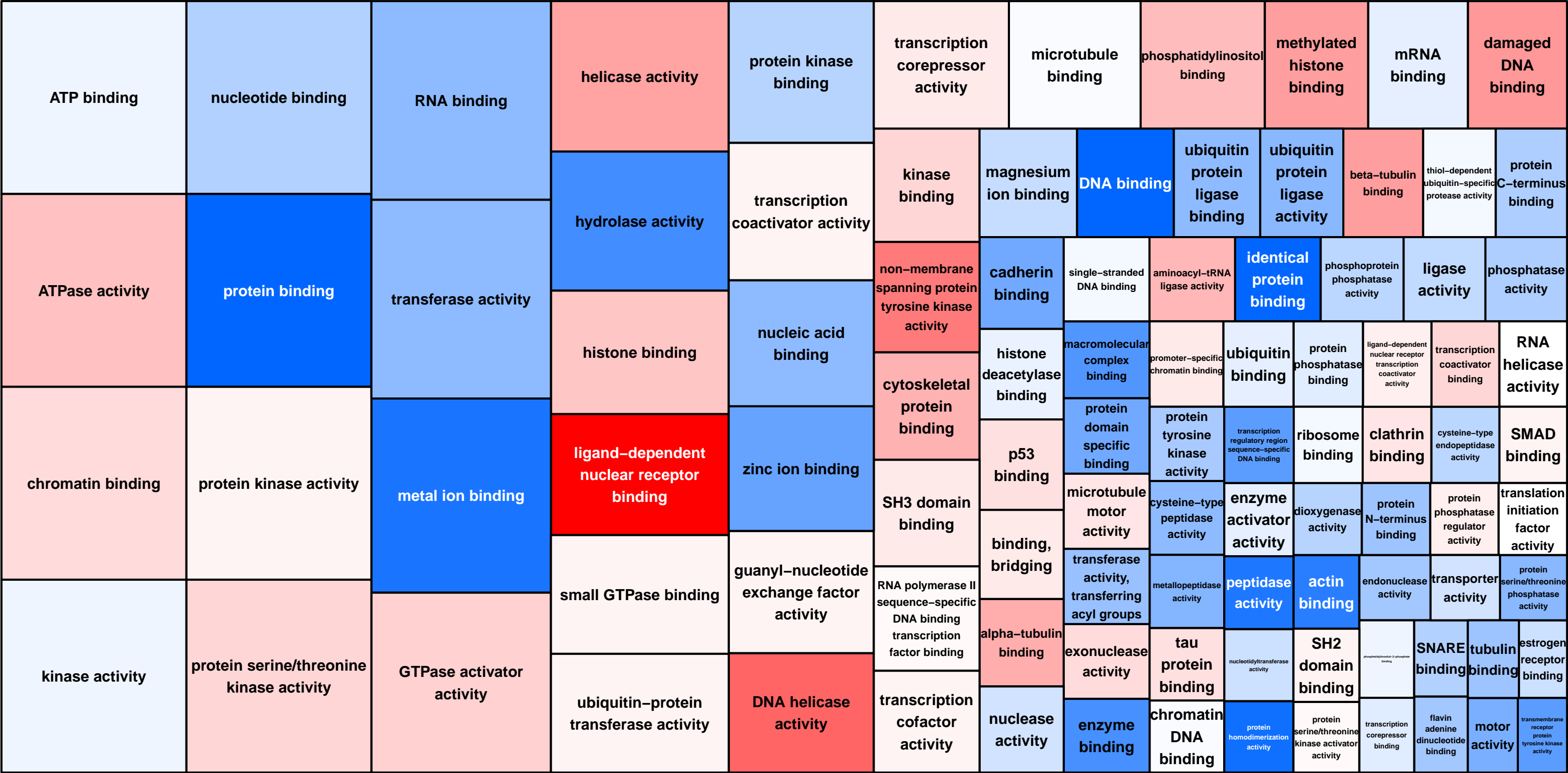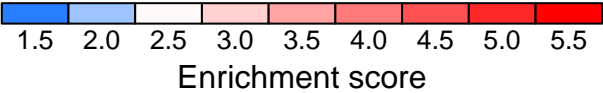

Supplement: Supplementary file 1 — Additional file 1: Figure S1. Structural and genomic features of circRNAs. Figure S2. Tree map of the enriched GO category (Biological Process, Molecular Function and Cellular Component) among the up-regulated genes for Follow up vs Baseline GEP-NET#1 comparison. Figure S3. Tree map of the enriched GO category (Biological Process, Molecular Function and Cellular Component) among the up-regulated genes for Follow up vs Baseline GEP-NET#4 comparison. Figure S4. Tree map of the enriched GO category (Biological Process, Molecular Function and Cellular Component) among the up-regulated genes for Follow up vs Baseline GEP-NET#5 comparison. Figure S5. Tree map of the enriched GO category (Biological Process, Molecular Function and Cellular Component) among the down-regulated genes for Follow up vs Baseline GEP-NET#1 comparison. Figure S6. Tree map of the enriched GO category (Biological Process, Molecular Function and Cellular Component) among the down-regulated genes for Follow up vs Baseline GEP-NET#4 comparison. Figure S7. Tree map of the enriched GO category (Biological Process, Molecular Function and Cellular Component) among the down-regulated genes for Follow up vs Baseline GEP-NET#5 comparison. Table S1. List of all circRNAs identified in the whole cohort and relative annotations. [file 12967_2023_4417_MOESM1_ESM.zip › Additional file/Figure S4.pdf]

Biological Processes

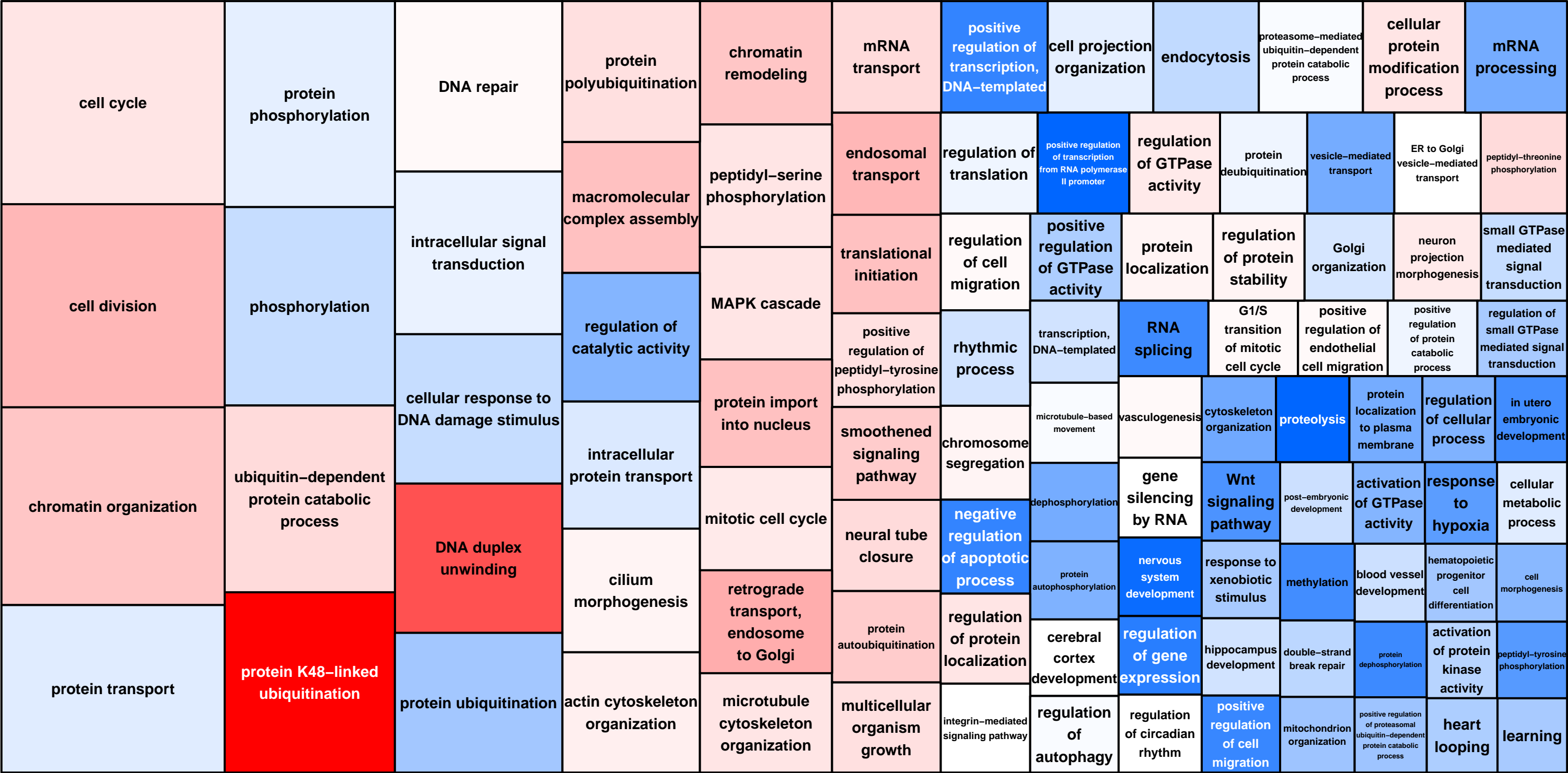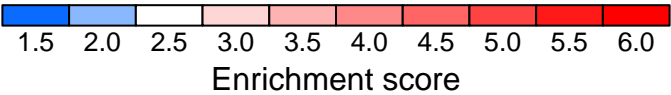

Molecular Functions

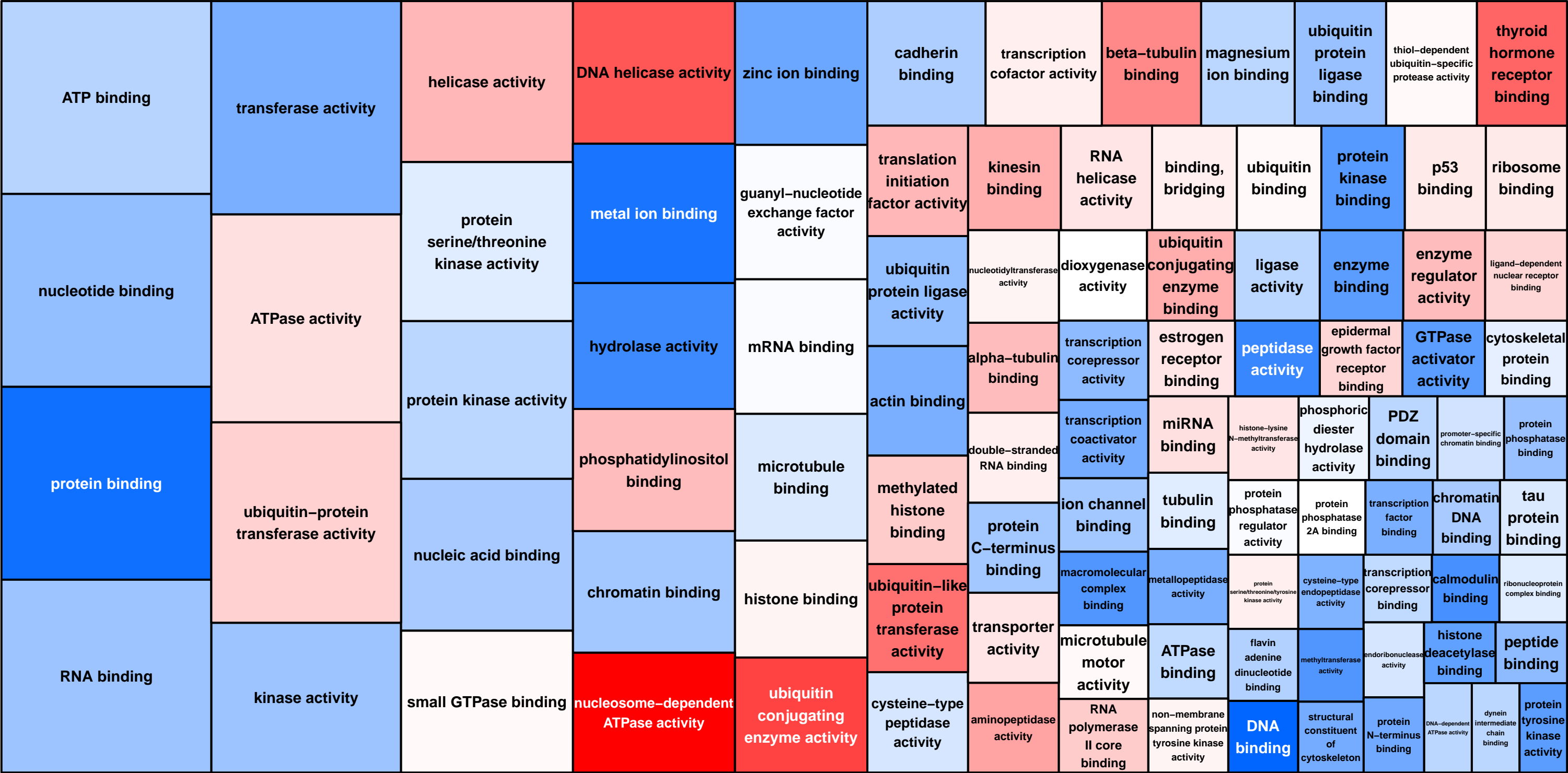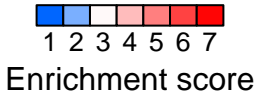

# Cellular Components

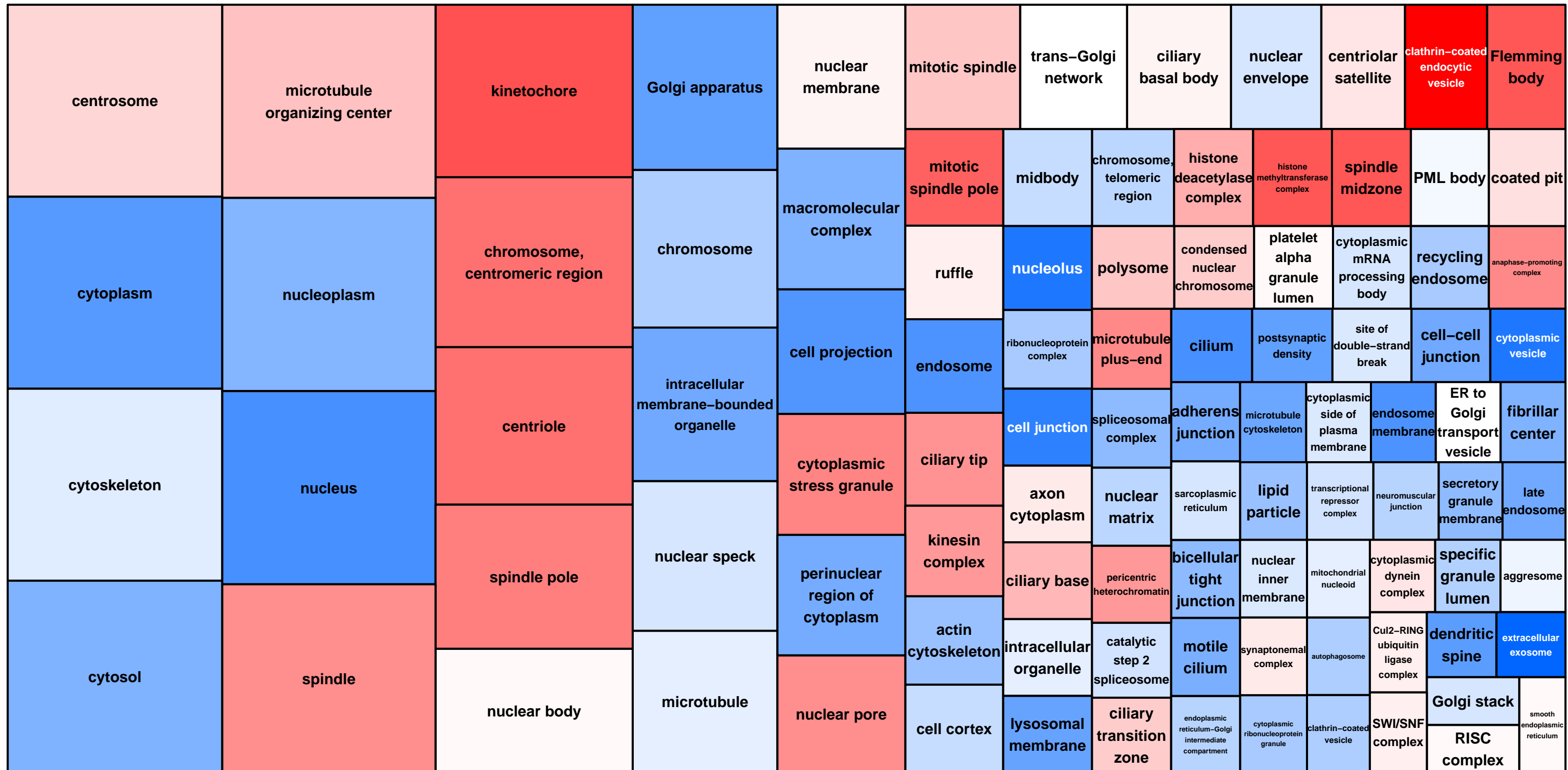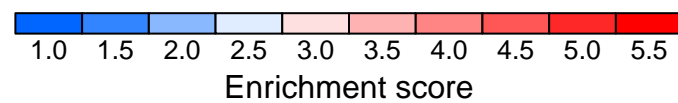

Supplement: Supplementary file 1 — Additional file 1: Figure S1. Structural and genomic features of circRNAs. Figure S2. Tree map of the enriched GO category (Biological Process, Molecular Function and Cellular Component) among the up-regulated genes for Follow up vs Baseline GEP-NET#1 comparison. Figure S3. Tree map of the enriched GO category (Biological Process, Molecular Function and Cellular Component) among the up-regulated genes for Follow up vs Baseline GEP-NET#4 comparison. Figure S4. Tree map of the enriched GO category (Biological Process, Molecular Function and Cellular Component) among the up-regulated genes for Follow up vs Baseline GEP-NET#5 comparison. Figure S5. Tree map of the enriched GO category (Biological Process, Molecular Function and Cellular Component) among the down-regulated genes for Follow up vs Baseline GEP-NET#1 comparison. Figure S6. Tree map of the enriched GO category (Biological Process, Molecular Function and Cellular Component) among the down-regulated genes for Follow up vs Baseline GEP-NET#4 comparison. Figure S7. Tree map of the enriched GO category (Biological Process, Molecular Function and Cellular Component) among the down-regulated genes for Follow up vs Baseline GEP-NET#5 comparison. Table S1. List of all circRNAs identified in the whole cohort and relative annotations. [file 12967_2023_4417_MOESM1_ESM.zip › Additional file/Figure S5.pdf]

## Biological Processes

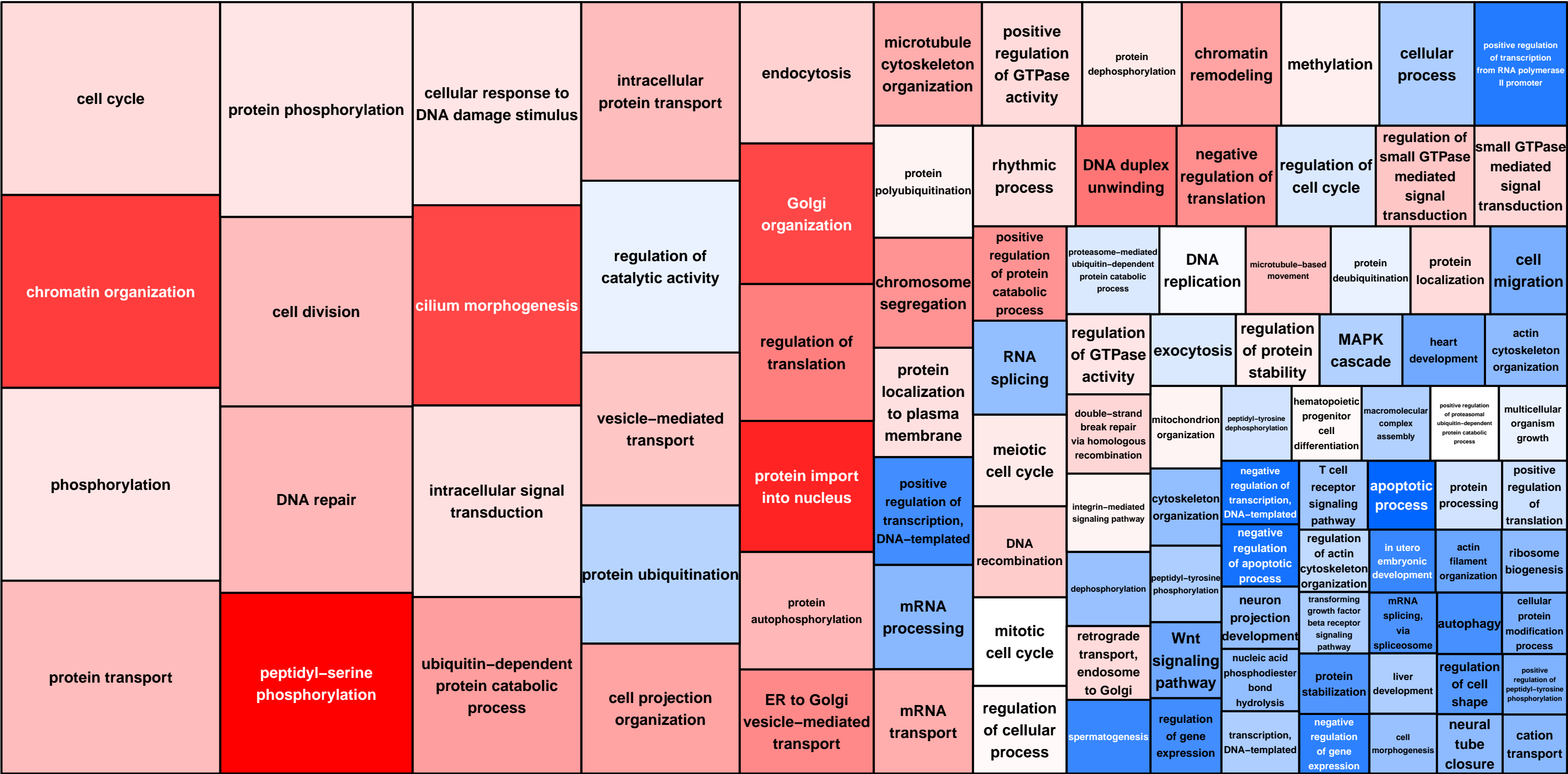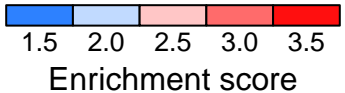

## Molecular Functions

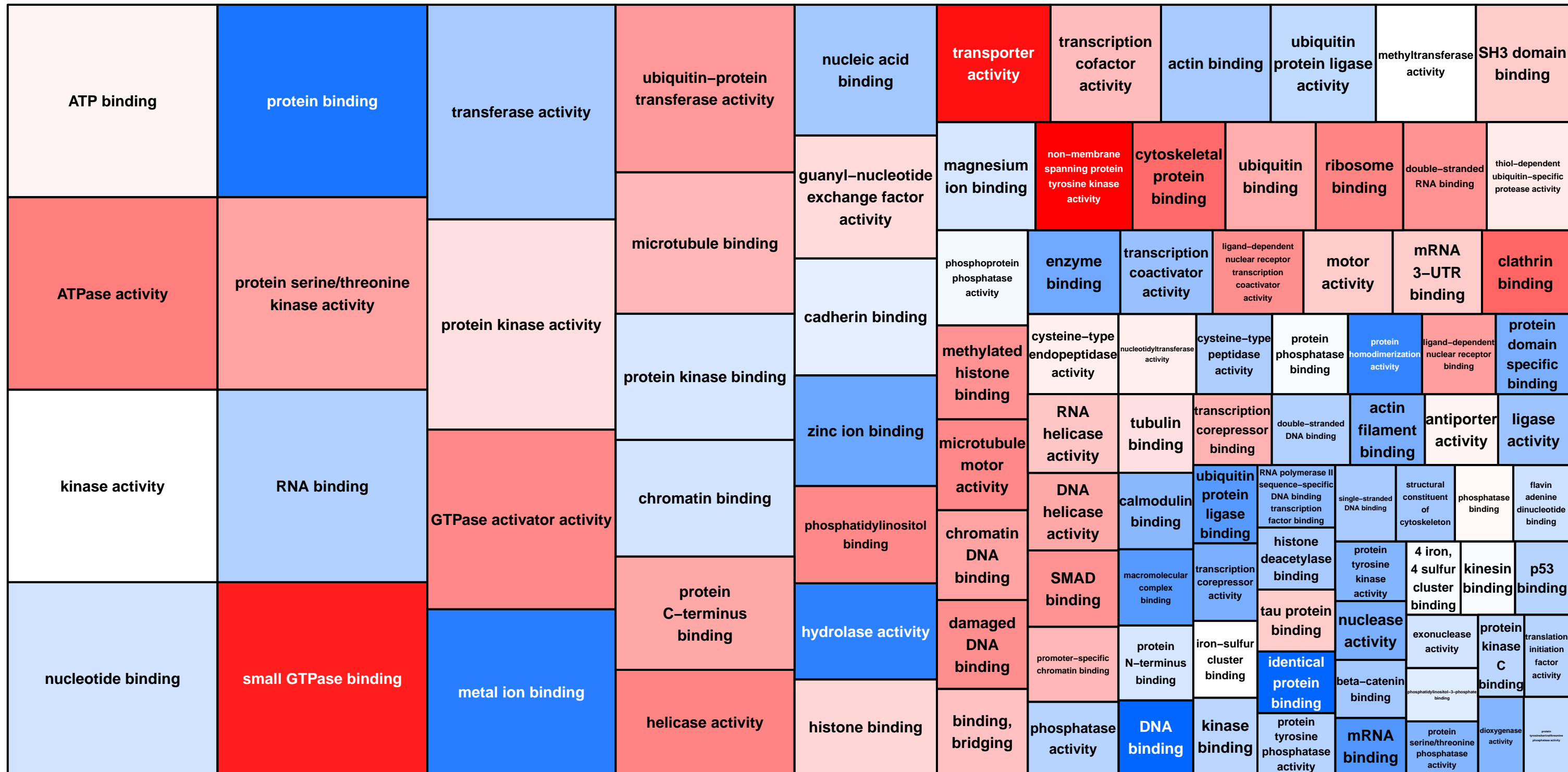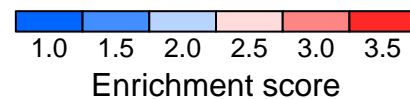

Cellular Components

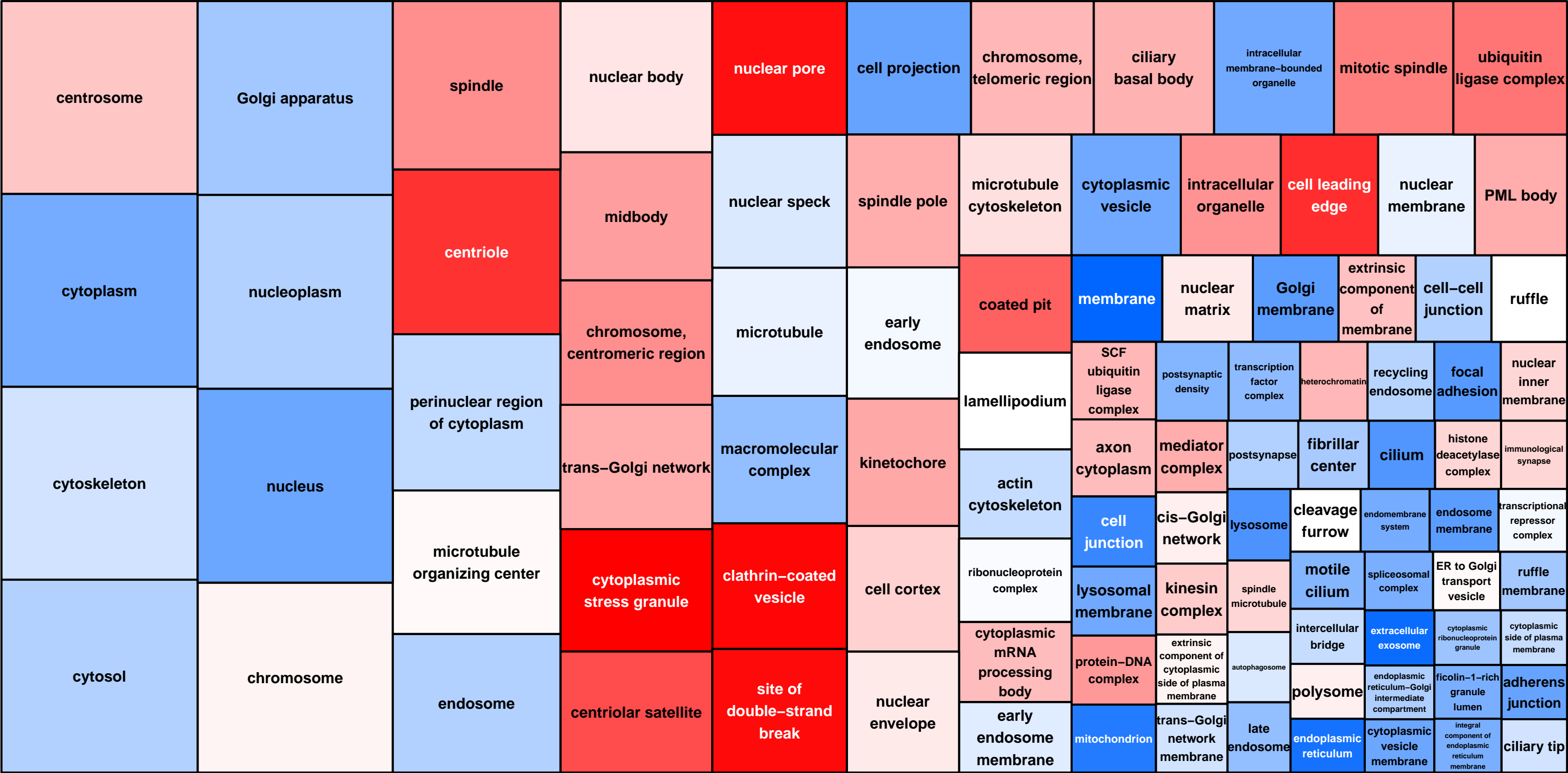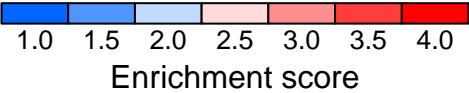

Supplement: Supplementary file 1 — Additional file 1: Figure S1. Structural and genomic features of circRNAs. Figure S2. Tree map of the enriched GO category (Biological Process, Molecular Function and Cellular Component) among the up-regulated genes for Follow up vs Baseline GEP-NET#1 comparison. Figure S3. Tree map of the enriched GO category (Biological Process, Molecular Function and Cellular Component) among the up-regulated genes for Follow up vs Baseline GEP-NET#4 comparison. Figure S4. Tree map of the enriched GO category (Biological Process, Molecular Function and Cellular Component) among the up-regulated genes for Follow up vs Baseline GEP-NET#5 comparison. Figure S5. Tree map of the enriched GO category (Biological Process, Molecular Function and Cellular Component) among the down-regulated genes for Follow up vs Baseline GEP-NET#1 comparison. Figure S6. Tree map of the enriched GO category (Biological Process, Molecular Function and Cellular Component) among the down-regulated genes for Follow up vs Baseline GEP-NET#4 comparison. Figure S7. Tree map of the enriched GO category (Biological Process, Molecular Function and Cellular Component) among the down-regulated genes for Follow up vs Baseline GEP-NET#5 comparison. Table S1. List of all circRNAs identified in the whole cohort and relative annotations. [file 12967_2023_4417_MOESM1_ESM.zip › Additional file/Figure S6.pdf]

Biological Processes

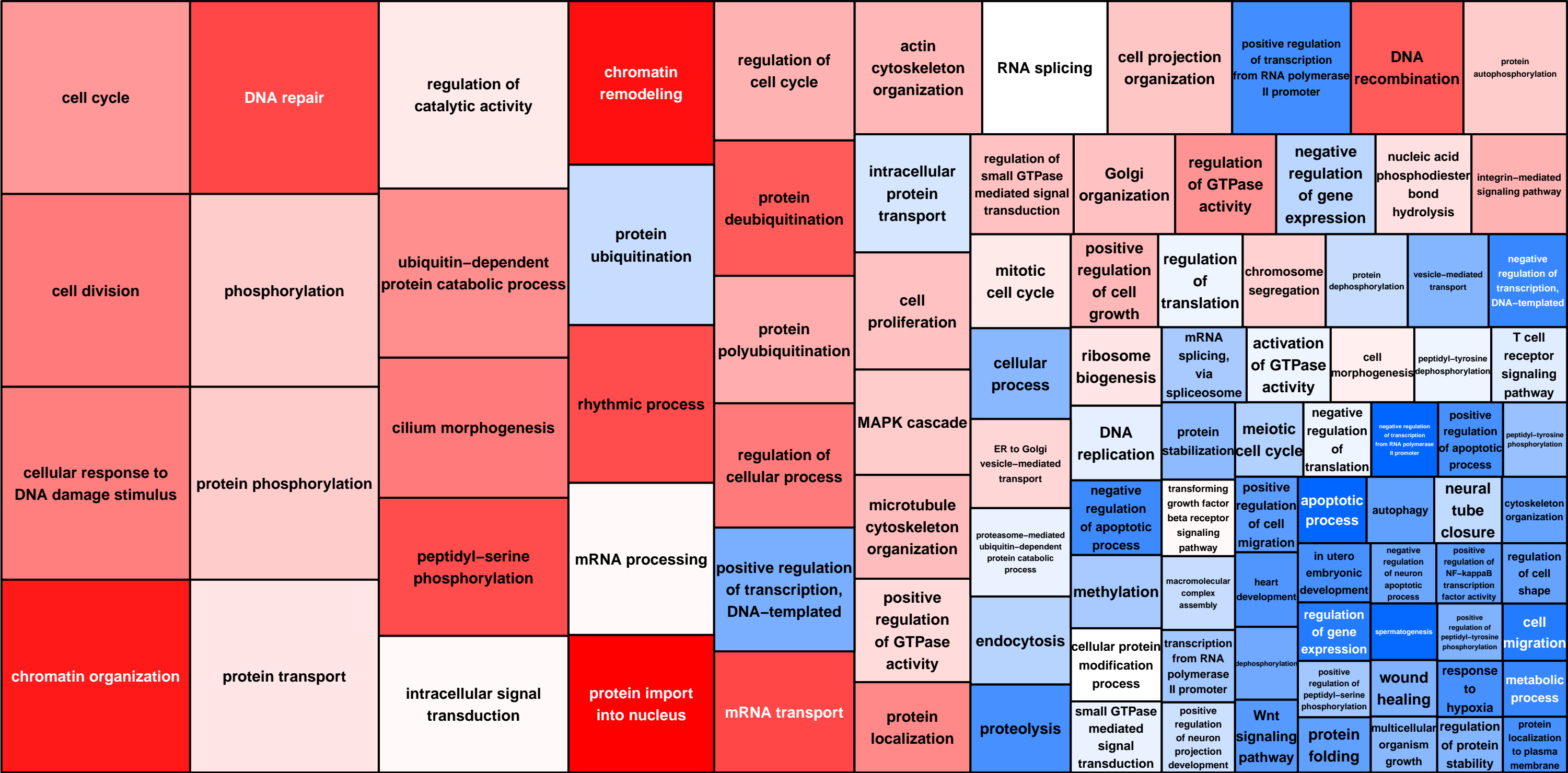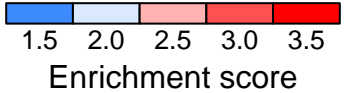

Molecular Functions

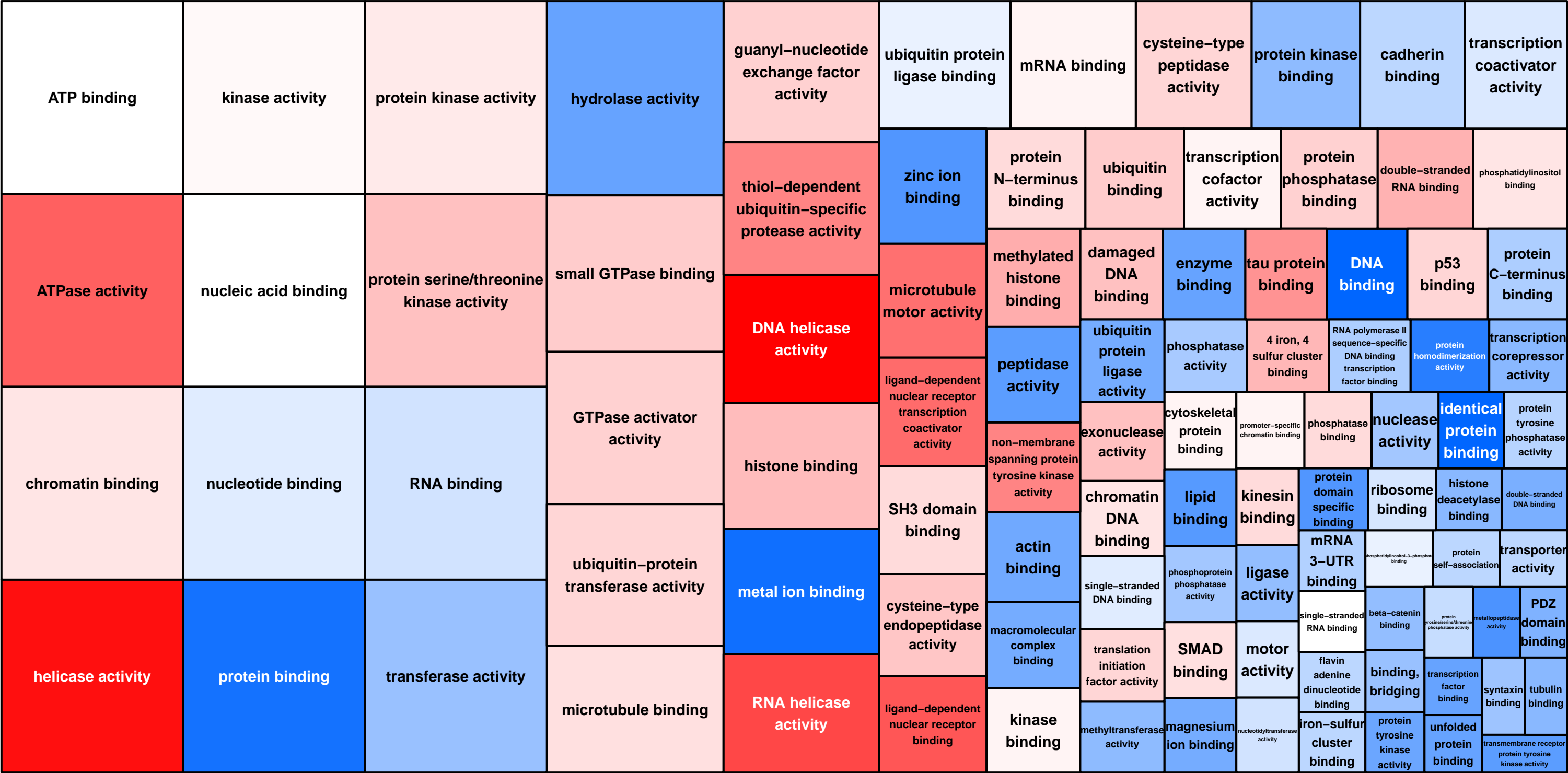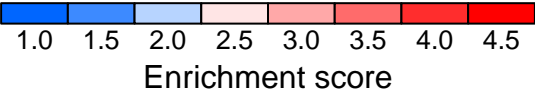

## Cellular Components

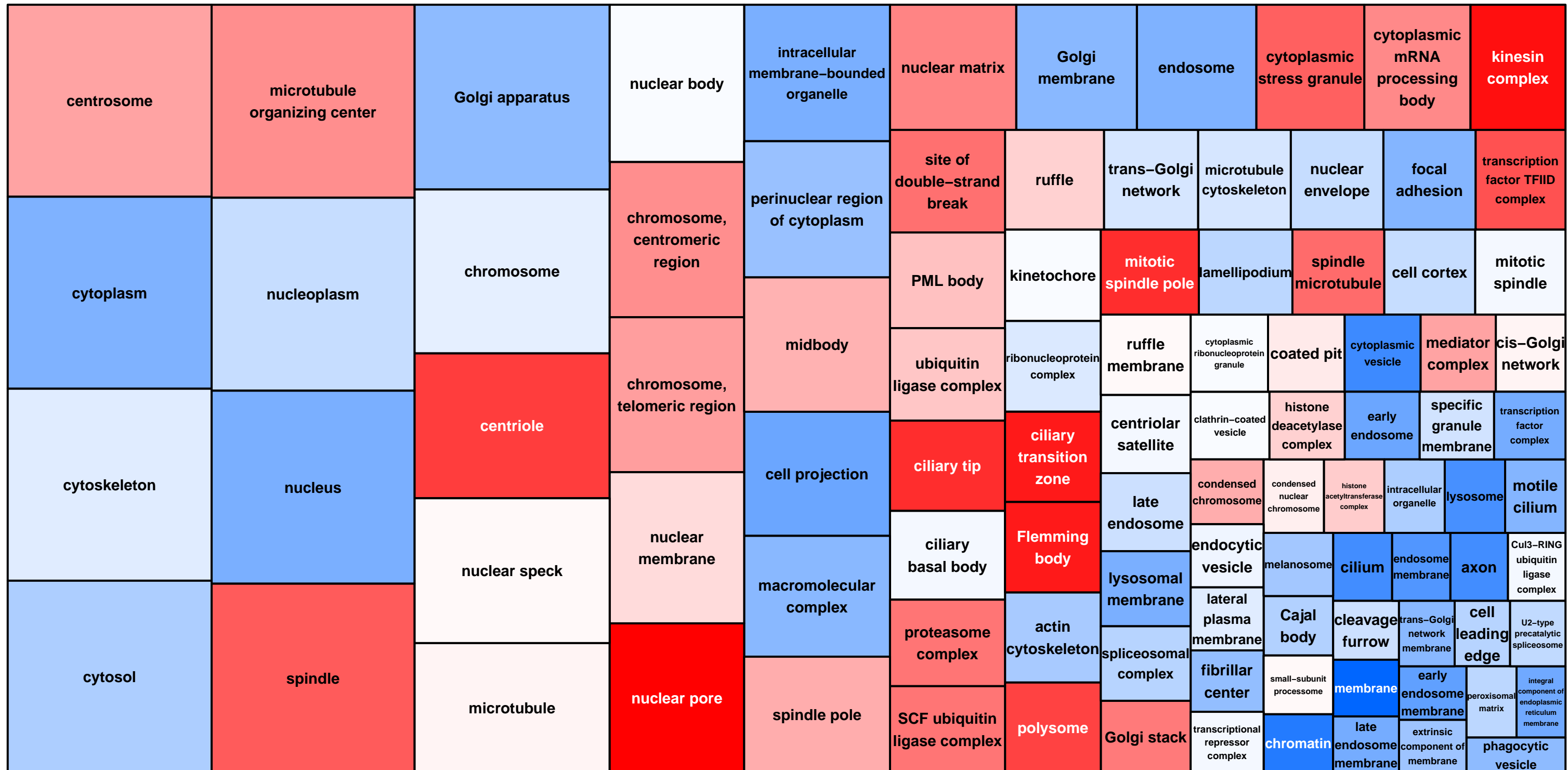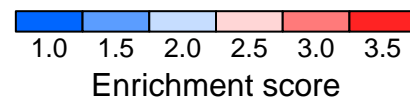

Supplement: Supplementary file 1 — Additional file 1: Figure S1. Structural and genomic features of circRNAs. Figure S2. Tree map of the enriched GO category (Biological Process, Molecular Function and Cellular Component) among the up-regulated genes for Follow up vs Baseline GEP-NET#1 comparison. Figure S3. Tree map of the enriched GO category (Biological Process, Molecular Function and Cellular Component) among the up-regulated genes for Follow up vs Baseline GEP-NET#4 comparison. Figure S4. Tree map of the enriched GO category (Biological Process, Molecular Function and Cellular Component) among the up-regulated genes for Follow up vs Baseline GEP-NET#5 comparison. Figure S5. Tree map of the enriched GO category (Biological Process, Molecular Function and Cellular Component) among the down-regulated genes for Follow up vs Baseline GEP-NET#1 comparison. Figure S6. Tree map of the enriched GO category (Biological Process, Molecular Function and Cellular Component) among the down-regulated genes for Follow up vs Baseline GEP-NET#4 comparison. Figure S7. Tree map of the enriched GO category (Biological Process, Molecular Function and Cellular Component) among the down-regulated genes for Follow up vs Baseline GEP-NET#5 comparison. Table S1. List of all circRNAs identified in the whole cohort and relative annotations. [file 12967_2023_4417_MOESM1_ESM.zip › Additional file/Figure S7.pdf]
